# PanoromiX: a time-course network medicine platform integrating molecular assays and pathophenotypic data

**DOI:** 10.1186/s12859-018-2494-6

**Published:** 2018-11-29

**Authors:** Ruoting Yang, Daniel Watson, Joshua Williams, Raina Kumar, Ross Campbell, Uma Mudunuri, Rasha Hammamieh, Marti Jett

**Affiliations:** 1 Advanced Biomedical Computational Science, Frederick National Laboratory for Cancer Research sponsored by the National Cancer Institute, Frederick, MD 21702-5010 USA; 2 Integrative Systems Biology Program, US Army Center for Environmental Health Research, 568 Doughten Drive, Fort Detrick, MD 21702-5010 USA

## Abstract

**Background:**

Network medicine aims to map molecular perturbations of any given diseases onto complex networks with functional interdependencies that underlie a pathological phenotype. Furthermore, investigating the time dimension of disease progression from a network perspective is key to gaining key insights to the disease process and to identify diagnostic or therapeutic targets. Existing platforms are ineffective to modularize the large complex systems into subgroups and consolidate heterogeneous data to web-based interactive animation.

**Results:**

We have developed PanoromiX platform, a data-agnostic dynamic interactive visualization web application, enables the visualization of outputs from genome based molecular assays onto modular and interactive networks that are correlated with any pathophenotypic data (MRI, Xray, behavioral, etc.) over a time course all in one pane. As a result, PanoromiX reveals the complex organizing principles that orchestrate a disease-pathology from a gene regulatory network (nodes, edges, hubs, etc.) perspective instead of snap shots of assays. Without extensive programming experience, users can design, share, and interpret their dynamic networks through the PanoromiX platform with rich built-in functionalities.

**Conclusions:**

This emergent tool of network medicine is the first to visualize the interconnectedness of tailored genome assays to pathological networks and phenotypes for cells or organisms in a data-agnostic manner. As an advanced network medicine tool, PanoromiX allows monitoring of panel of biomarker perturbations over the progression of diseases, disease classification based on changing network modules that corresponds to specific patho-phenotype as opposed to clinical symptoms, systematic exploration of complex molecular interactions and distinct disease states via regulatory network changes, and the discovery of novel diagnostic and therapeutic targets.

**Electronic supplementary material:**

The online version of this article (10.1186/s12859-018-2494-6) contains supplementary material, which is available to authorized users.

## Background

Many diagnostic and therapeutic target discovery research studies are rapidly adopting a temporal systems biology design that combines multi-population, multi-tissue, and multi-omics. Many believe a disease is a disturbance enacted on the “driver” genes that lead to a cascade of changes of other genes: initially to their first-degree interaction neighbors, followed by downstream effects [1]. Ideally, the network can be seen as several molecular modules that can be activated sequentially from one to the other. For example, Hwang et al. summarized a prion progression genetic network using seven mouse strains [2]. Huang et al. used gene expression profiling to show that trajectories of neutrophil differentiation converge to common networks eventually although the perturbations originated from different networks [3].

Visually tracing temporal changes emerging from biological processes has a high value in light of the molecular ontology and clinical observations. However, most current network platforms (e.g. Cytoscape [4], Gephi [5], IPA [6]), tools (e.g.VisANT [7], PATIKA [8], NAViGaTOR [9], GeneVis [10], yfiles [11], VANTED [12], Cancer PanorOmics [13]), custom R codes [14] generate static snapshots, which are inadequate at consolidating large-scale heterogeneous data. Even for advanced users, making a dynamic modular network is very labor intensive, requiring knowledge of several toolboxes and custom coding.

Multi-omics time series network analysis has three major challenges:

### Modularity

Components with similar functions need to be tightened, labelled, and moved together.

### Interactivity

Multiple layers of heterogeneous information need to be visually distinguished. Animation, search, zoom, and basic functions that modify the characteristics of components on site must be integrated.

### Sharability

The network model needs to be easily shared, restored, and compared, without installing software.

## Implementation

PanoromiX is hosted online at https://bioinfo-abcc.ncifcrf.gov/panoromics/. It is written in PhP5 and open-source Javascript libraries D3.js, jquery.js, and dat.gui.js. Since the PanoromiX software application is completely web-based, there are no installation requirements and no restrictions on which operating systems can be used. The software can be launched on any computer system that is connected to the internet and capable of running one of the current web browser applications with JavaScript capabilities enabled (Internet Explorer, Google Chrome, Mozilla Firefox, Safari).

## Results

The PanoromiX application accepts data in tab-delimited text files with the file extension *.txt*. PanoromiX requires a “node” file containing information about individual points (nodes) of interest that will be plotted as part of the network visualization. A “link” file containing information about links or relationships between each of these points (nodes) is optional.

PanoromiX uses standard node-edge design to denote molecules and their interactions, respectively. The node shape and fill color can represent molecule types and expression levels. For example, one can display a longitudinal study of cancer patients where a node reprents a patient, illness stages as different colors, and therapies as shape . PanoromiX requires an input text file to initially populate the node of a biological network. This node file defines the nodes and requires an ID and Color for each designated node. Additional biological group information and optional graphical descriptions can be provided. An optional edge file defines network edges through a list of connected Source and Target IDs. The detailed description of the characteristics of node and edge file is listed in Tables 1 and 2. After submitting simple text files of network attributes, users can easily design and display an interactive network online using the toolbar.

**Table 1:** PanoromiX Node file

Data Field	Data Type	Required	Description
id	General text	Required	This field denotes the unique id of this node or point. It is used by the application to reference each node individually and perform operations such as linking and defining colors, shape and so on. This field must be unique.
name	General text	Required	This field is the label that is displayed under each node on the visualization and although similar to the id in our example data, this label can be whatever the user chooses, and in addition does not need to be a unique value.
group	General text	Required	Since the PanoromiX application creates modules or groups from the data it processes, this field is necessary to indicate which group, or module, a particular node is a member of. This field does not need to be unique as many nodes may belong to the same group.
type	General text	Required	This field is used to denote a characteristic about each node, aside from its group. For example, one can define both ‘Male’ and ‘Female’ nodes.
description	General Text	Optional	This field allows the user to enter a short paragraph of text *(256 characters or less)* containing a description, or additional information related to each node. We will discuss how this is displayed when we illustrate how to interact with the visualization.
size	Numerical	Optional	Depending on the nature of your data, it may be beneficial to render some nodes larger on the screen, and some smaller. This numerical value (1–5) allows you to do just that. It controls the dimensions or size of each node on screen.
color	Numerical	Optional	This field allows the user to enter a numerical value (1–5) to display a time-point profile associated with the node.
shape	Numerical	Optional	This field allows the user to specify a general shape for each of the nodes by using a numerical value (0–5) in this column. 0 – circle, 1 – square, 2 – diamond, 3 – cross, 4 - triangle (downward pointing), 5 – triangle (upward pointing)

**Table 2:** PanoromiX Edge file

Data Field	Data Type	Required	Description
sourceId	General text	Required	This field must contain the id value of the node *(from your corresponding nodes file)* where the link will be drawn from. If you enter an invalid node id here the link will be ignored.
targetId	General text	Required	This field must contain the id value of the node *(from your corresponding nodes file)* where the link will be drawn to. If you enter an invalid node id here the link will be ignored.
link_scale	Numerical	Optional	The user can enter the width of a link
marker_start	Numerical	Optional	The user can indicate the style of the marker that is drawn at the start of the link using a value (0–3). 0 – circle, 1 – square, 2 – arrow, 3 - stub
marker_end	Numerical	Optional	The user can indicate the style of the marker that is drawn at the end of the link using a value (0–3). 0 – circle, 1 – square, 2 – arrow, 3 - stub
linkName	General text	Optional	The user can enter the name of a link
linkColor	Numerical	Optional	The user can enter the color of a link

### Modularity

**Fig. 1:**
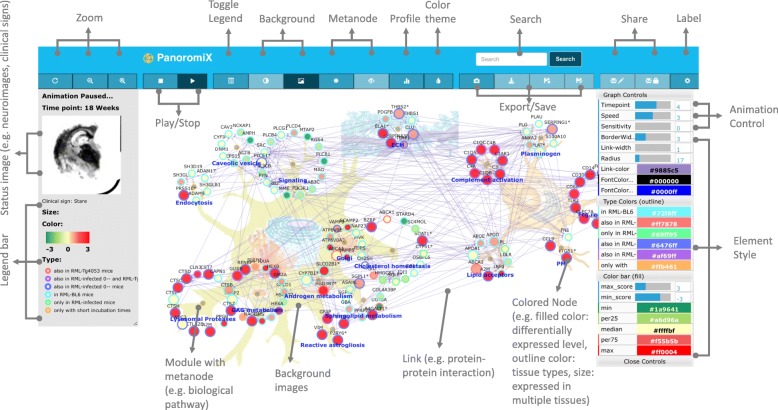
An illustration of prion accumulation network. (Full animation is available on the PanoromiX website)

PanoromiX has a special meta-node design that allows user to define modules (“group” function in Table 1). All the components belonging to a module connect to its meta-node (Fig. 1). One can move the entire module by its meta-node. In addition, the users can also upload icons that will represent meta-nodes. The icons need to be .jpeg files and image dimension or resolution is not restricted as long as they are not greater than 5 MB in size each.

### Interactivity

PanoromiX has a full interactive toolbar with search, zoom, animation play/stop, theme change, save, download, share functions, and a time point labelling tool. On the right hand side, there is a control panel to adjust animation speed, link width, font color, as well as other useful functions including sensitivity filtering. The sensitivity filtering feature allows the users to see only the node with desired changes between two adjacent time points.

PanoromiX also has a unique dynamic legend panel to associate the network model with external resources, such clinical variables, images, behavioral observations. (Additional files 1, 2 and 3) The node shape outline color is defined as a ‘type’ (Table 1), which is also displayed as a link on the legend panel. Clicking the link highlights all nodes of the same type. The users can employ this attribute to refer to any external knowledge, such as tissue-specific, disease/drug-related, ethnicity/gender-specific information, and even correlations to clinical variables. Furthermore, users can upload an associated image, such as a histology or MRI image, and insert a description for each time point. Figure 1 shows such an example, where the user has uploaded brain images and behavior descriptions corresponding to prion disease severity in mice, while the six types of nodes underneath the image panel represent the six mouse-prion strains used in the study.

### Sharability and scalability

In an avenue comparable to how Jupyter notebooks [15] and R markdown [16] enable the sharability and reproducibility of modelling, PanoromiX also allows users to build and deposit their network models as templates to a permanent web space where other researchers can freely download and reproduce their results. The cloud-sourcing of network models significantly enhances the opportunity for collaboration between researchers, and will drastically reduce the manual labor required for designing visual representations. The users can designate the model to be read-only or writable. In read-only mode, the user can still modify the network and input their own contributions, but not overwrite the original work.

**Fig. 2:**
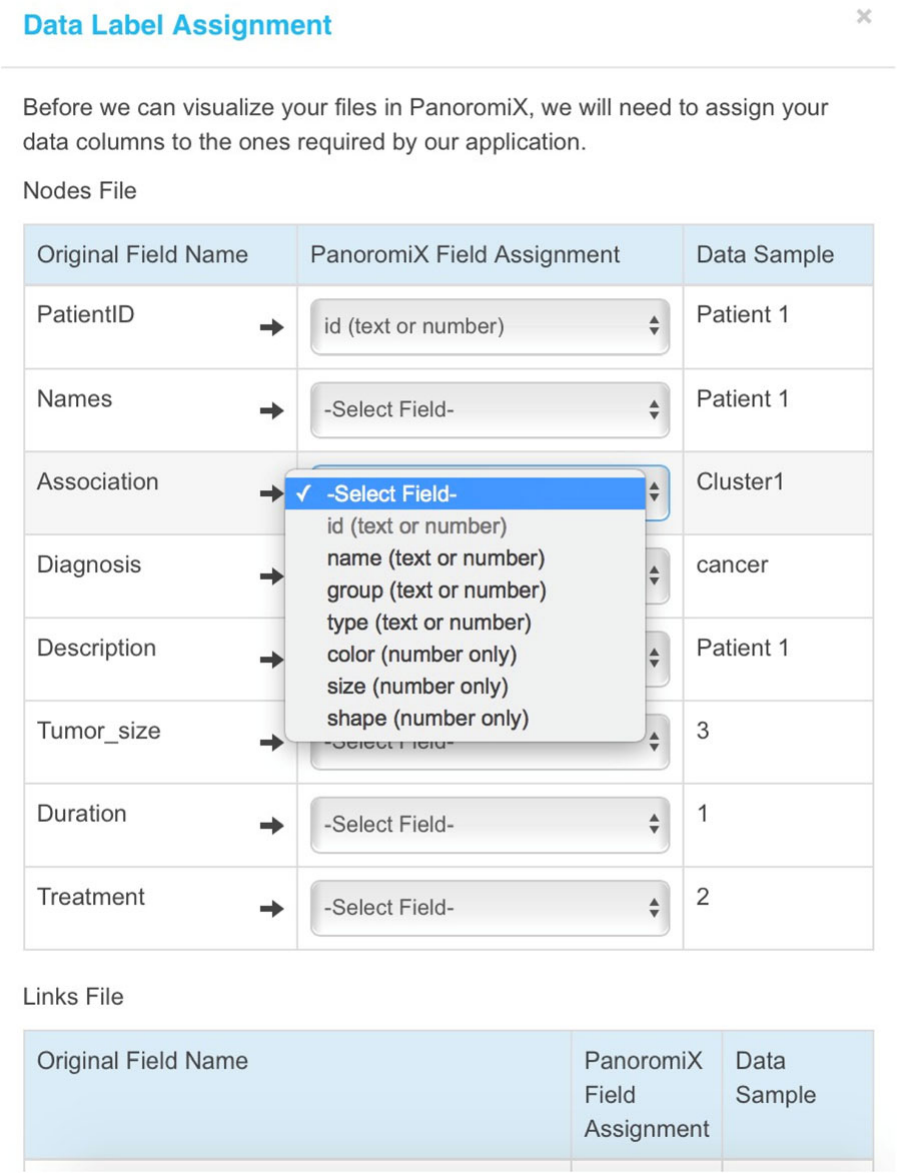
Data Label Assignment to import the network files from Cytoscape and IPA

PanoromiX also supports imported network files from Cytoscape [4], IPA [6], and custom files. Once the files are uploaded, a “Data Label Assignment” page will pop up (Fig. 2) for the user to indicate which PanoromiX data fields will correspond to the columns in the Cytoscape/IPA data files. The visualization module of PanoromiX is written by Javascript. However, the computational module in the backend server can call Python and R scripts, whenever more advanced computational techniques are needed. The output will be stored in a text file that the Java script can read and parse. By hosting these computations on a powerful remote server, PanoromiX alleviates the problem of visualizing massive networks on small workspaces, and helps make large-scale network analysis available to scientists without extensive high-performance computing capabilities. In comparion to the popular tools like Cytoscape [4], Gephi [5], and IPA [6], PanoromiX has unique and robust features. Particularly valuable is PanoromiX’ interactive animation, which is very helpful in exploring and presenting time-series trends (Table 3, Additional file 4).

**Table 3:** Comparison of PanoromiX to other major network packages

Name	web-based design, display, and share	Time-series interactive animation	Support Metanode/Module	Temporal profile analysis	Reproducibility	Learning curve	Open Source
Panoromix	web-based, full interactivity	Playing mode with highlight, annotation, search, filtering	Yes	Sentivity filtering; display progfiles	web-based model depository	No software intallation, no programming needed	Free
Cytoscape	desktop version and Cytoscape.js	Non-interactive videos using DynNetwork	Yes	Sensitivity filtering; display profiles	share session files	Need to install; need to learn plugins; extensive programming required for Cytoscape.js	Free
Gephi	desktop	Non-interactive videos	Metanode pugin	No	share session files	Need to install; need to learn plugins	Free
IPA	Web-based	No	No	No	share session files	Need toinstall; no programming required	Commercial
R packages	Shiny server	Non-interactive videos using animation + igraph	No	Statistical Packages	R Notebook/R Markdown	Need to install R and many packages; Extensive programming needed	Shiny server (have commercial version)
VisANT	Web-based	No	Yes	No	web sharing	Need to install, no programming needed	Free
yED	desktop and yED live	No	No	No	share session files	No software installation, no programming needed	Free
VANTED	desktop	No	No	display profiles	share excel files	Need to install, no programming needed	Free
NAViGaTOR	desktop	No	No	display profiles	share text files	currently unsupported, no programming needed	Free
PATIKA	web-based	No	Yes	display profiles	share text file	currently unsupported, no programming needed	Free
Cancer PanorOmics	web-based	No	No	No	share text file	No software installation, no programming needed	Free
GENeVis	desktop	No	No	display profiles	share text file	Need to install, no programming needed	Free

### Limitation and future work

Displaying huge-interconnected networks on the computer or smart device with limited memory is a universal challenge. The computational complexity of force-directed graph in D3.js is O(nlog n), thus rending 5000 nodes and edges will expect a slowdown. For very large networks, offline static network tools like Gephi [5] will be more practical. In the future, we will implete more analytic components, including network reconstruction algotihms to automatically create network modules.

## Availability and requirements

PanoromiX is publicly available at https://bioinfo-abcc.ncifcrf.gov/panoromics/

Operating systems: Windows/OSX.

Programming language: PHP and Javascript.

Browsers: IE 9, Firefox 31, Chrome 31, Safari 5.1, Opera 24, Opera Mini 8, iOS safari 7.1, Android Browser 4.4, or later.

## Conclusions

PanoromiX is a comprehensive platform to enhance network model reproducibility, time-series interpretability, and data associability. This emergent tool of network medicine visualizes the interconnectedness of tailored genome assays to pathological networks and phenotypes for cells or organisms in a data-agnostic manner. As an advanced network medicine tool, PanoromiX allows monitoring of panel of biomarker perturbations over the progression of diseases, disease classification based on changing network modules that corresponds to specific patho-phenotype as opposed to clinical symptoms, systematic exploration of complex molecular interactions and distinct disease states via regulatory network changes, and the discovery of novel diagnostic and therapeutic targets.

## Additional files

Additional file 1: Illustrative videos. (MP4 4756 kb)

Additional file 2: Illustrative videos. (MP4 4563 kb)

Additional file 3: Illustrative videos. (MP4 3415 kb)

Additional file 4: User Guide (DOCX 4721 kb)

## Abbreviations

IPA: Ingenuity Pathway Analysis
MRI: Magnetic Resonance Imaging

## References

[1] Beyer A, Bandyopadhyay S, Ideker T (2007). Integrating physical and genetic maps: from genomes to interaction networks. Nat Rev Genet.

[2] Hwang D, Lee IY, Yoo H, Gehlenborg N, Cho JH, Petritis B, Baxter D, Pitstick R, Young R, Spicer D (2009). A systems approach to prion disease. Mol Syst Biol.

[3] Huang S, Eichler G, Bar-Yam Y, Ingber DE (2005). Cell fates as high-dimensional attractor states of a complex gene regulatory network. Phys Rev Lett.

[4] Shannon P, Markiel A, Ozier O, Baliga NS, Wang JT, Ramage D, Amin N, Schwikowski B, Ideker T (2003). Cytoscape: a software environment for integrated models of biomolecular interaction networks. Genome Res.

[5] Bastian M, Heymann S, Jacomy M (2009). Gephi: an open source software for exploring and manipulating networks. Icwsm.

[6] Ingenuity Pathways Analysis software web link. [http://www.ingenuity.com/].

[7] Hu Z, Mellor J, Wu J, DeLisi C (2004). VisANT: an online visualization and analysis tool for biological interaction data. BMC bioinformatics.

[8] Dogrusoz U, Erson E, Giral E, Demir E, Babur O, Cetintas A, Colak R (2005). PATIKA web: a web interface for analyzing biological pathways through advanced querying and visualization. Bioinformatics.

[9] Brown KR, Otasek D, Ali M, McGuffin MJ, Xie W, Devani B, ILv T, Jurisica I (2009). NAViGaTOR: network analysis, visualization and graphing Toronto. Bioinformatics.

[10] Baker C, Carpendale MT, Prusinkiewicz P, Surette MG: GeneVis: visualization tools for genetic regulatory network dynamics. In: *Proceedings of the conference on Visualization'02: 2002*. IEEE Computer Society: 243–250.

[11] Wiese R, Eiglsperger M, Kaufmann M. Yfiles—visualization and automatic layout of graphs. In: Graph drawing software: Springer; 2004. p. 173–91.

[12] Junker BH, Klukas C, Schreiber F (2006). VANTED: a system for advanced data analysis and visualization in the context of biological networks. BMC bioinformatics.

[13] Mateo L, Guitart-Pla O, Pons C, Duran-Frigola M, Mosca R, Aloy P (2017). A PanorOmic view of personal cancer genomes. Nucleic Acids Res.

[14] Team RC: R language definition (2000). Vienna, Austria: R foundation for statistical computing.

[15] Kluyver T, Ragan-Kelley B, Pérez F, Granger BE, Bussonnier M, Frederic J, Kelley K, Hamrick JB, Grout J, Corlay S (2016). Jupyter notebooks-a publishing format for reproducible computational workflows. ELPUB.

[16] Baumer B, Cetinkaya-Rundel M, Bray A, Loi L, Horton NJ (2014). R markdown: integrating a reproducible analysis tool into introductory statistics. *arXiv preprint arXiv:14021894*.

