# Supplementary material for: PanoromiX: a time-course network medicine platform integrating molecular assays and pathophenotypic data
Source: BMC Bioinformatics. 2018 Nov 29;19:458. doi: 10.1186/s12859-018-2494-6 (PMC6267067; doi:10.1186/s12859-018-2494-6)
Supplement: Supplementary file 4 — User Guide (DOCX 4721 kb) [file 12859_2018_2494_MOESM4_ESM.docx]

**
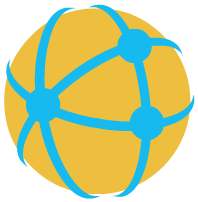
**PanoromiX User’s Guide

**December 2016**

**Version 2.0**


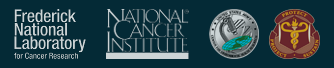

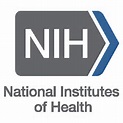


**Contents**

[Chapter 1: Introduction 1](#_Toc469406055)

[Scope and Purpose 1](#_Toc469406056)

[Using the PanoromiX Software 1](#_Toc469406057)

[Chapter 2: Creating a PanoromiX Project 2](#_Toc469406058)

[Preparing Data Files and Optional Icons 2](#_Toc469406059)

[Understanding and Creating the Required Nodes File 2](#_Toc469406060)

[Understanding and Creating the Optional Links File 4](#_Toc469406061)

[Saving Optional Icon Images for Groups 5](#_Toc469406062)

[Importing Project Files through the PanoromiX Interface 6](#_Toc469406063)

[Selecting and Previewing Project Files 6](#_Toc469406064)

[Selecting and Previewing the Links File 8](#_Toc469406065)

[Selecting and Previewing the Custom Image File(s) 9](#_Toc469406066)

[Viewing Your Upload Report 11](#_Toc469406067)

[Configuring the Import 12](#_Toc469406068)

[Saving the Configuration and Importing the Files 14](#_Toc469406069)

[Viewing and Interacting with the Visualization 15](#_Toc469406070)

[The PanoromiX Network Visualization 15](#_Toc469406071)

[The PanoromiX Toolbar Controls 18](#_Toc469406072)

[The PanoromiX Dropdown Controls 22](#_Toc469406073)

[Customizing, Saving, Sharing, and Exporting Your Visualization 24](#_Toc469406074)

[Using the PanoromiX Controls to Customize Our Network 24](#_Toc469406075)

[Chapter 3: Advanced Features of PanoromiX 30](#_Toc469406076)

[Uploading a Previously Saved PanoromiX Project 30](#_Toc469406077)

[Including Time-Point Data for Animations in Your Project 31](#_Toc469406078)

[Including Additional Information: Node Shapes, Descriptions 32](#_Toc469406079)

[Chapter 4: Frequently Asked Questions and Troubleshooting 34](#_Toc469406080)

[Frequently Asked Questions 34](#_Toc469406081)

[What format does my data need to be in for the PanoromiX application? 34](#_Toc469406082)

[What hardware/software do I need to install to run the PanoromiX application? 34](#_Toc469406083)

[How can I prepare my images for uploading as icons? 34](#_Toc469406084)

[How can I share my PanoromiX project with a collaborator? 34](#_Toc469406085)

[How can I export my project as an image for publication or printing? 35](#_Toc469406086)

[Can I save my project and return to it at a later time to continue my work? 35](#_Toc469406087)

[Troubleshooting 35](#_Toc469406088)

[I am receiving errors while uploading my data, what should I do? 35](#_Toc469406089)

[I am having difficulty with some of the application options, or some options are not working for me. 35](#_Toc469406090)

[One of my recipients is having difficulty opening a shared link to my project. 35](#_Toc469406091)

[My icon images are not being displayed. What can I do? 36](#_Toc469406092)

[I have uploaded my data but the visualization canvas is blank and does not display my network. What can I do? 36](#_Toc469406093)

**Document Revisions**

| Date | Version Number | Document Changes |
| --- | --- | --- |
| 11/10/2015 | 0.1 | Initial Draft |
| 11/30/2015 | 0.2 | Initial Release |
| 08/15/2016 | 0.3 | Rebranding Update |
| 12/16/2016 | 0.4 | Updated Draft |

**DISCLAIMERS:**

The authors disclaim no conflict of interest. Research was conducted in compliance with all Federal Requirements. The views expressed are those of the authors and do not constitute endorsement by the U.S. Army.

Chapter 1: Introduction

## Scope and Purpose

PanoromiX is a novel data-driven web application for network visualization. It enables users to define a modularized, multi-layered network, and seamlessly display it as an interactive figure across most web browsers, by uploading their data in a text file. The user can customize many attributes of the network, and share interactive results easily via email. With minimal software installation and programming knowledge requirements, PanoromiX allows users to easily design, explore, and share informative, interactive networks.

The purpose of this guide is to equip the user of the PanoromiX application with all the direction they will need to successfully create, upload, and visualize their data through the web-based software interface. The document instructs the user on downloading and working with example data templates, creating new data files, uploading data and custom images through the PanoromiX interface, interacting with the network visualization, customizing the network and interface, and advanced customization options for PanoromiX.

## Using the PanoromiX Software

Since the PanoromiX software application is completely web-based, there are no installation requirements and no restriction on which operating system can be used. The software can be launched on any computer system that is connected to the internet and capable of running one of the current web browser applications with JavaScript capabilities enabled (Internet Explorer, Google Chrome, Mozilla Firefox, Safari). No programming or database knowledge is necessary; all functions that a user must perform are illustrated in this guide.

Here is the typical workflow involved in using PanoromiX for your visualizations:

1. Prepare your data files (and optional icons). More information is on page 2.
2. Upload your files through the PanoromiX interface. More information is on page 6.
3. View your upload report. More information is on page 11.
4. View and interact with the visualization. More information is on page 15.
5. Customize, save, share and export your visualization. More information is on page 24.

Chapter 2: Creating a PanoromiX Project

The PanoromiX application accepts data in tab-delimited text files with the file extension *.txt*. Many different applications exist to create files of this format but for the purpose of our user’s guide, we will illustrate the creation of data files using Microsoft Excel (2013) for Windows and Microsoft Excel (2011) for Mac. *(These specific versions are not required. The exact menu appearance and steps may vary from what is shown here. Please consult the user’s guide for your specific version if necessary.)*

In addition, you can also upload icons that will represent certain groups within your visualization and these need to be images *(less than 5MB each)* and formatted as jpeg with the file extension ‘jpg’. These can be created or saved from any source and image dimensions or resolution are not restricted as long as they are not greater than 5MB in size each. In the following steps we will explain and illustrate in detail how all of these files are prepared.

## Preparing Data Files and Optional Icons

Example: Using MS Excel 2013 (PC) & MS Excel 2011 (Mac)

The PanoromiX application accepts two data files that it processes to create the network visualization:

- A *required* **nodes** file containing information about individual points (nodes) of interest that will be plotted as part of your network visualization. More information about the structure and content of this file follows on this page.
- An *optional* **links** file which contains information about links or relationships between each of these points (nodes). More information about the structure and content of this file is on page 4.

In addition to these files, you can upload optional image icons to represent groups or modules from your data. Unlike the nodes and links files, which can be named according to your preference, the image icons must be of type jpeg (*.jpg*) and named *exactly* after the name of the group they are representing. More information about the image icons is on page 5.

### Understanding and Creating the Required Nodes File

Example: Creating a new nodes file using the template provided on the PanoromiX website

1. Download the example PanoromiX nodes file template from the Tutorial section of our website. It should be called: **PanoromiX-nodes-template.txt**
2. Open the file using MS Excel (examples in this guide use MS Excel 2013 – PC).
   The Text Import Wizard, Step 1 of 3 appears.
3. Select **Delimited** and then click **Next**.
   The Text Import Wizard, Step 2 of 3 appears.
4. Ensure that only the box next to **Tab** is checked. Click **Next**.
   The Text Import Wizard, Step 3 of 3 appears.
5. Select **General**, and then click **Finish**.
   The nodes template opens in Excel.
   The following table explains each field of the data and how it is used.

Table 1. PanoromiX Nodes Template

| Data Field | Data Type | Required | Description |
| --- | --- | --- | --- |
| **id** | General text | Required | This field denotes the unique id of this node or point. It is used by the application to reference each node individually and perform operations such as linking and defining colors, shape and so on.  **This field must be unique.** |
| **name** | General text | Required | This field is the label that is displayed under each node on the visualization and although similar to the id in our example data, this label can be whatever the user chooses, and in addition does not need to be a unique value. |
| **group** | General text | Required | Since the PanoromiX application creates modules or groups from the data it processes, this field is necessary to indicate which group, or module, a particular node is a member of. This field does not need to be unique as many nodes may belong to the same group. |
| **type** | General text | Required | This field is used to denote a characteristic about each node, aside from its group. For example, in our data set, we have defined both ‘Active’ and ‘Inactive’ nodes. This allows the visualization to display more than just groups for your nodes, it also allows you to indicate whether they are all the same type, or whether they are different. For a different data set, this could just as easily be ‘Male’ and ‘Female’, where both could reside in the same group, but have different gender. |
| **description** | General Text | Optional | This field allows the user to enter a short paragraph of text *(256 characters or less)* containing a description, or additional information related to each node. We will discuss how this is displayed when we illustrate how to interact with the visualization. |
| **size** | Numerical | Optional | Depending on the nature of your data, it may be beneficial to render some nodes larger on the screen, and some smaller. This numerical value (1-5) allows you to do just that. It controls the dimensions or size of each node on screen. |
| **color** | Numerical | Optional | This field allows the user to enter a numerical value (1-5) to display a time-point profile associated with the node. |
| **shape** | Numerical | Optional | This field allows the user to specify a general shape for each of the nodes by using a numerical value (0-5) in this column.  0 – circle, 1 – square, 2 – diamond, 3 – cross, 4 - triangle (downward pointing), 5 – triangle (upward pointing) |

1. Enter your data into the template as described and once you are finished, save as a tab-delimited text file (*.txt*). You may choose any name for this file as long as you are able to distinguish it as your nodes file for PanoromiX. Please pay careful attention below to the differences between performing this step on Windows and Mac. We will save ours as *nodes.txt*.

NOTE: On the Windows version of MS Excel, you will be saving as Text (tab-delimited) (*.txt) and on the Mac version of MS Excel you will be saving the file as Windows Formatted Text (.txt).

### Understanding and Creating the Optional Links File

Example: Creating a new links file using the provided template from the PanoromiX website

1. Download the example PanoromiX links file template from the tutorial section of our website. It should be called: **PanoromiX-links-template.txt**.
2. Open the file using MS Excel (examples in this guide use MS Excel 2013 – PC).
   The Text Import Wizard, Step 1 of 3 appears.
3. Select **Delimited** and then click **Next**.
   The Text Import Wizard, Step 2 of 3 appears.
4. Ensure that only the box next to **Tab** is checked. Click **Next**.
   The Text Import Wizard, Step 3 of 3 appears.
5. Select **General**, and then click **Finish**.
   The links file template opens in Excel.
   The following table explains each field of the data and how it is used.

Table 2. PanoromiX Links Template

| Data Field | Data Type | Required | Description |
| --- | --- | --- | --- |
| **sourceId** | General text | Required | This field must contain the id value of the node *(from your corresponding nodes file)* where the link will be drawn from. If you enter an invalid node id here the link will be ignored. |
| **targetId** | General text | Required | This field must contain the id value of the node *(from your corresponding nodes file)* where the link will be drawn to. If you enter an invalid node id here the link will be ignored. |
| **link_scale** | Numerical | Optional | The user can enter the width of a link |
| **marker_start** | Numerical | Optional | The user can indicate the style of the marker that is drawn at the start of the link using a value (0-3).  0 – circle, 1 – square, 2 – arrow, 3 - stub |
| **marker_end** | Numerical | Optional | The user can indicate the style of the marker that is drawn at the end of the link using a value (0-3).  0 – circle, 1 – square, 2 – arrow, 3 - stub |
| **linkName** | General text | Optional | The user can enter the name of a link |
| **linkColor** | Numerical | Optional | The user can enter the color of a link |

1. Enter your data into the template as described and once you are finished, save as a tab delimited text file (*.txt*). You may choose any name for this file as long as you are able to distinguish it as your nodes file for PanoromiX. Please pay careful attention below to the differences between performing this step on Windows and Mac. We will save ours as *links.txt*.

NOTE: On the Windows version of MS Excel, you will be saving as Text (tab delimited) (*.txt) and on the Mac version of MS Excel you will be saving the file as Windows Formatted Text (.txt).

### Saving Optional Icon Images for Groups

Example: Saving new image icons for our example data groups

The nodes file data has three distinct groups: Group A, Group B, and Group C.

1. Open or rename the desired jpeg image you want to use as a group icon and save it with the filename corresponding to the group you want it to represent. Only use the image file extension *.jpg*.
2. We will save three jpeg images to represent our groups. These three files will take the names: *Group A.jpg*, *Group B.jpg*, and *Group C.jpg*.

NOTE: The filename of each image must be *exactly* that of the group it is meant to represent, and end with the file extension *.jpg*. Any differences in this and your icons may not display as intended.

## Importing Project Files through the PanoromiX Interface

Example: Uploading the example files (IPA_nodes.txt, IPA_links.txt, Cytoscape-nodes.txt, and Cytoscape-links.txt)

Once you have prepared your data and image files, it is time to upload them to the application where they will be scanned and evaluated before being processed for visualization. If there are any errors in the formatting of your data, the application will indicate this on your upload report. You can either continue if the error will not cause a software failure, or fix the error and resubmit your files for processing. The process for selecting a file and uploading it follows.

### Selecting and Previewing Project Files

Example: Selecting and previewing the project files

In this procedure, we import the sample files, *IPA_nodes.txt* or *IPA_links.txt*. The sample *Cytoscape-nodes.txt* and *Cytoscape-links.txt* files are also available for import.

1. Navigate to the PanoromiX home page:

- Click the **Get Started** menu at the top of the home page.
  or
- Click the
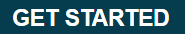
 after the sample graphic on the home page.

The Get Started: Create a Project page appears with three steps for uploading your data and creating a visualization.

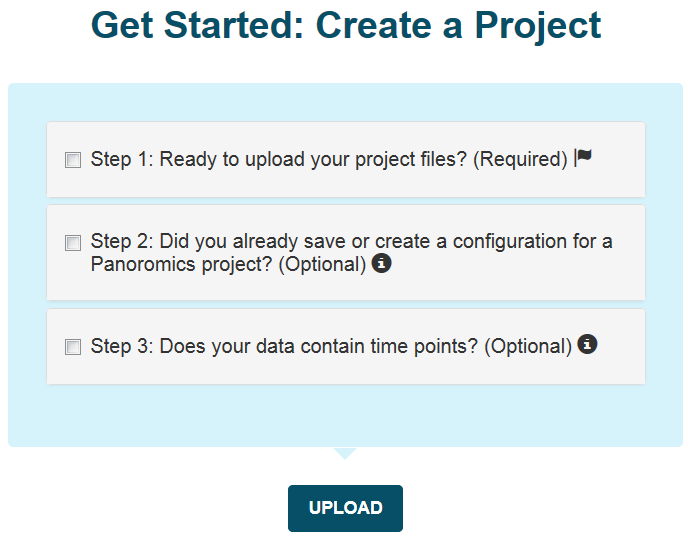


1. Check the **Step 1. Ready to upload your project files? (Required)** box.

**NOTE:** For our sample, leave Steps 2 and 3 cleared. **Step 2** uploads a configuration file from a previously saved PanoromiX project, a process that is explained in “Uploading a Previously Saved PanoromiX Project” on page 30. **Step 3** is for uploading a complex set of data that includes multiple layers of information for the purposes of animating graphical changes to your network visualization. This is for advanced users and discussed later.

1. Once you check **Step 1**, Step 1 expands.

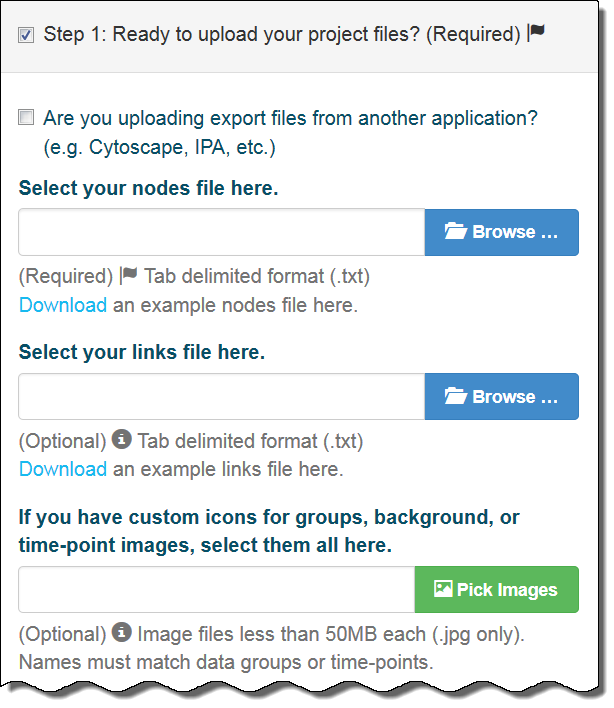

2. Check **Are you uploading export files from another application? (for example, Cytoscape, IPA, etc.)**.

**NOTE**: You can download an example nodes file by clicking **Download an example nodes file here**. The node file is required and must be in tab delimited format with the file extension (*.txt*).

1. Below the *Select your nodes file here* label, click
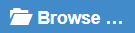
 and navigate to the *ipa_nodes.txt* file on your file system.

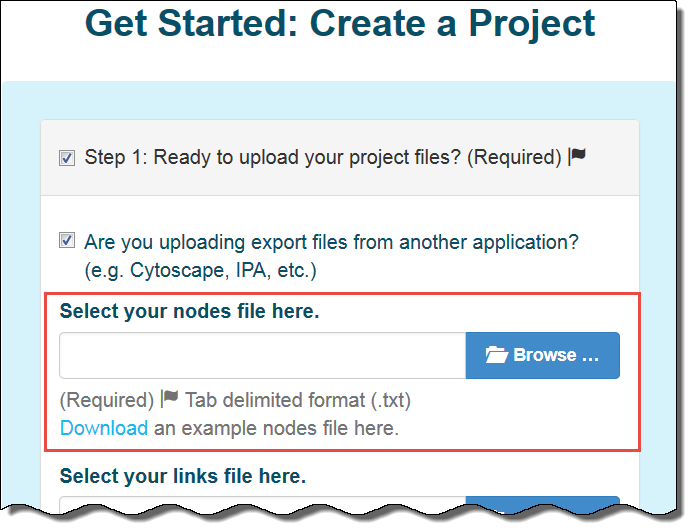

   A file preview window appears.
2. Click the magnifier to display the complete file in a popup window; click the X to close the popup.
3. If you need to select another file, click
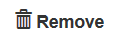
 and the file is removed *(from the interface – not your hard disk)*. Browse for another file.
4. If you do not want to import a links file or custom icon images, you can scroll to the bottom of the page and click
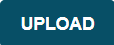
. Otherwise, proceed to the following section, “Selecting and Previewing the Links File.”

### Selecting and Previewing the Links File

Example: Selecting and previewing the links file we created, links.txt

1. On the Get Started: Create a Project page, under the Step 1 check box and nodes file information, you can import a links file.

**NOTE**: You can download an example links file by clicking **Download an example links file here**. The links file is optional and must be in tab-delimited format with the file extension (*.txt*).

1. To import a links file, under the **Select your links file here** label, click
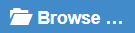
 and select the *ipa_links.txt* file.

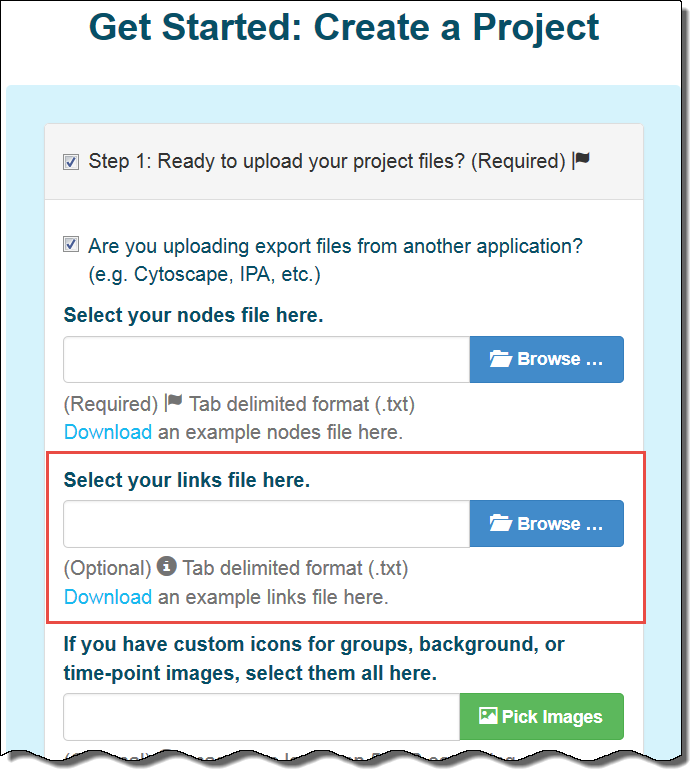

   A file preview window appears.
2. Click the magnifier to display the complete file in a popup window, and click **X** to close the popup.
3. If you need to select another file, click
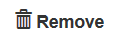
 and the file is removed *(from the interface – not your hard disk)*. Browse for another file.
4. If you do not want to import custom icon images, scroll to the bottom of the page and click
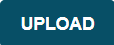
. Otherwise, proceed to the following section, “Selecting and Previewing the Custom Image File(s).”

### Selecting and Previewing the Custom Image File(s)

Example: Selecting and previewing the image icons files we created, Group A.jpg, Group B.jpg, Group C.jpg.

**NOTE**: Icon image files must be less than 50MB each (*.jpg* only). The names of the files must match data groups or time-points.

1. On the Get Started: Create a Project page, click
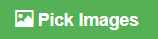
 and select one or more *.jpg* files.

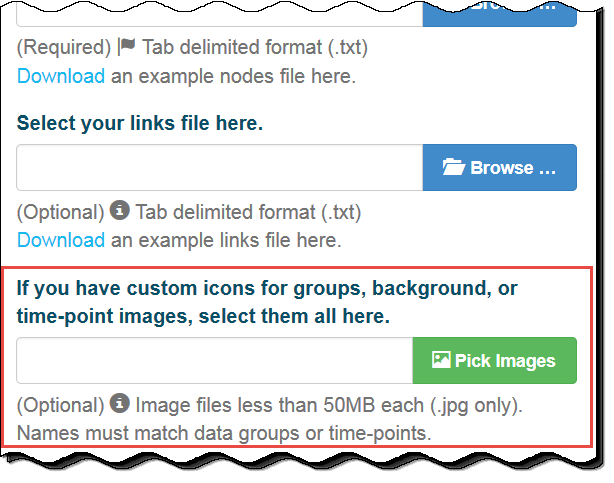

   A preview window appears, displaying the selected icons.
2. To remove the icons, click
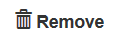
and all the icons are removed at once *(from the interface – not your hard disk)*. Pick more files.
3. Once the icon images are selected, scroll to the bottom of the page and click
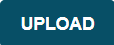
.
   The File Upload Report appears.

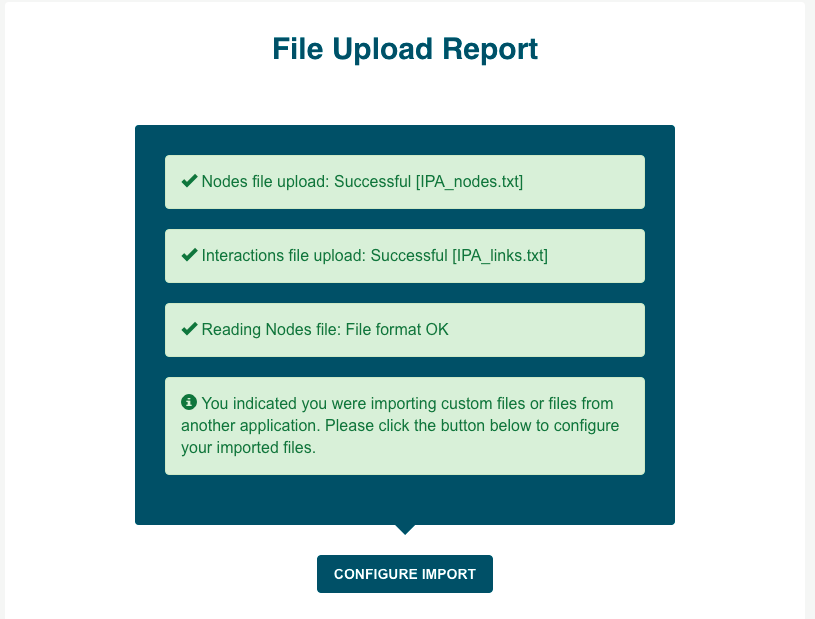

4. Click
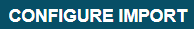
. Proceed to “Configuring the Import” on page 12 for information about how to configure the imported files.

## Viewing Your Upload Report

Example: Understanding the upload report after uploading the example files

Once you have uploaded your data files and optional custom images to the PanoromiX application, an upload report appears that informs you of the status of your files and allows you to proceed with the visualization, or else allows you to correct errors if any have been discovered.

As long as the data we have uploaded is formatted correctly, this success message indicates that we have successfully uploaded our nodes file to the application.


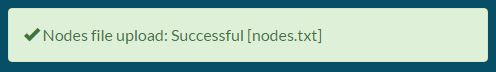


If you upload a links file for your project as in this example*,* the following success message simply reports that it has been successfully uploaded to the application. Since this file is optional, this message is only displayed if you upload a links file.


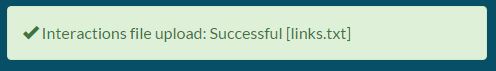


The next message regarding the node file shown below indicates that the nodes file is in the correct format, and the PanoromiX application has been able to convert your information into the format required to produce the network visualization.


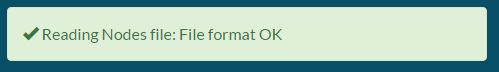


The next message(s) indicates that our custom icon images have been successfully uploaded as well. In our case, we have uploaded 3 of them, and they are listed here:


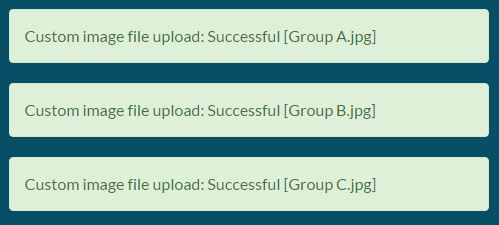


## Configuring the Import

Example: Indicate what each column in the file represents in PanoromiX

At this stage you indicate which columns in your data files you want to use to represent your data in PanoromiX. A table showing the nodes file (and links if uploaded) is presented. You may select each data column and assign it separately.


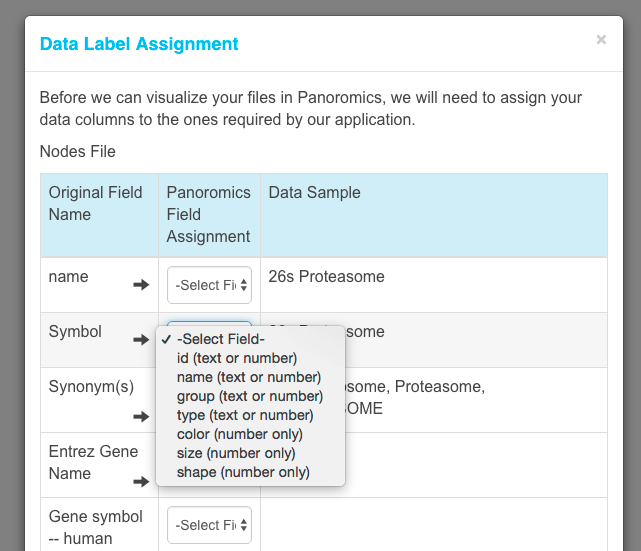


**NOTE**: It is not necessary to assign every field to successfully import files into PanoromiX.

The following sections show how to configure the sample IPA and Cytoscape node and link files.

For the sample IPA_nodes.txt and IPA_links.txt files

Configure the *ipa_nodes.txt* file as follows:

| IPA Links File Original Field Name | PanoromiX Field Name |
| --- | --- |
| name | Name |
| Symbol | Id |
| Exp Log Ratio | Color |
| Location | Type |
| Family | group |

Configure the *ipa_links.txt* file as follows:

| IPA Links File Original Field Name | PanoromiX Field Name |
| --- | --- |
| From Molecule(s) | sourceId |
| To Molecule(s) | targetId |

For the sample Cytoscape-nodes.txt and Cytoscape-links.txt files

Configure the *Cytoscape-nodes.txt* file as follows:

**NOTE**: Multiple values indicate optional field assignments.

| Cytoscape Nodes Original Field Name | PanoromiX Field Name |
| --- | --- |
| name / shared name | id |
| name / shared name | name |
| nodeFillColor_aaa / pValue_aaa / adjustedPValue_aaa | color |
| nodeType_aaa | type |
| xx_aaa / cluster | group |

Configure the *Cytoscape-links.txt* file as follows:

| Cytoscape Links Original Field Name | PanoromiX Field Name |
| --- | --- |
| From Molecule(s) | sourceId |
| To Molecule(s) | targetId |

## Saving the Configuration and Importing the Files

1. Once you are done, click
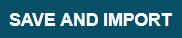
 to save your changes. Click
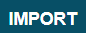
 to process your files.


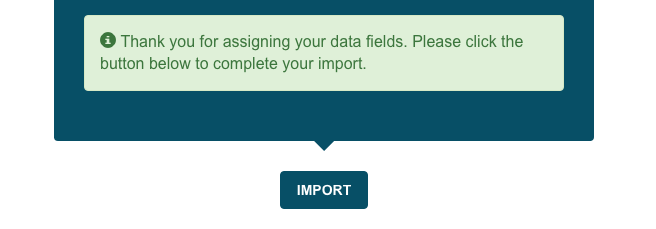


**NOTE**: If there are errors after the import, continue to Troubleshooting on page 35.

1. If there are no errors, the File Upload Report displays the status “Import Complete.” To view the imported information, click
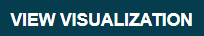
.


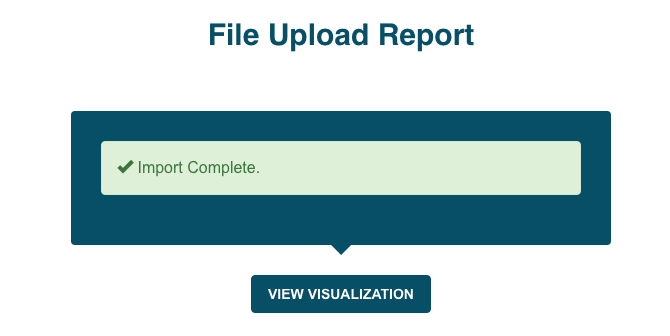


Your results appear.


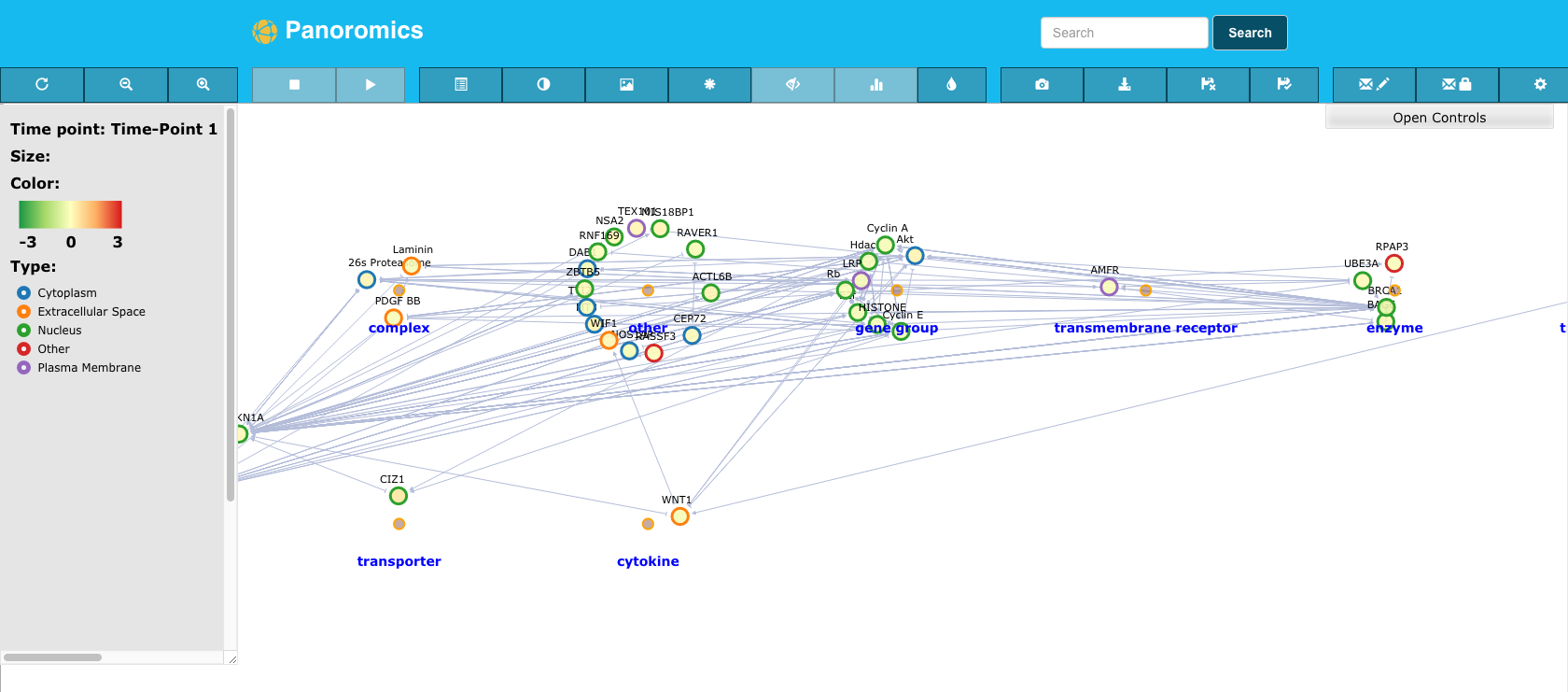


## Viewing and Interacting with the Visualization

Example: Network visualization generated from the sample data

This section outlines and identifies the main network visualization interface, and identifies the controls and various options that are provided to the user for customizing and interacting with the graph. In the below example, we see the result of our data set and custom image icons that were uploaded in the previous sections:

### The PanoromiX Network Visualization

By clicking with your mouse (or touching the screen for some devices), there are a number of different activities and options that can be activated depending on the area that you click, or the specific type of click you apply *(double-click, click and drag, etc.)*. As we identify each component of the display, we will also describe any associated actions which may be performed.


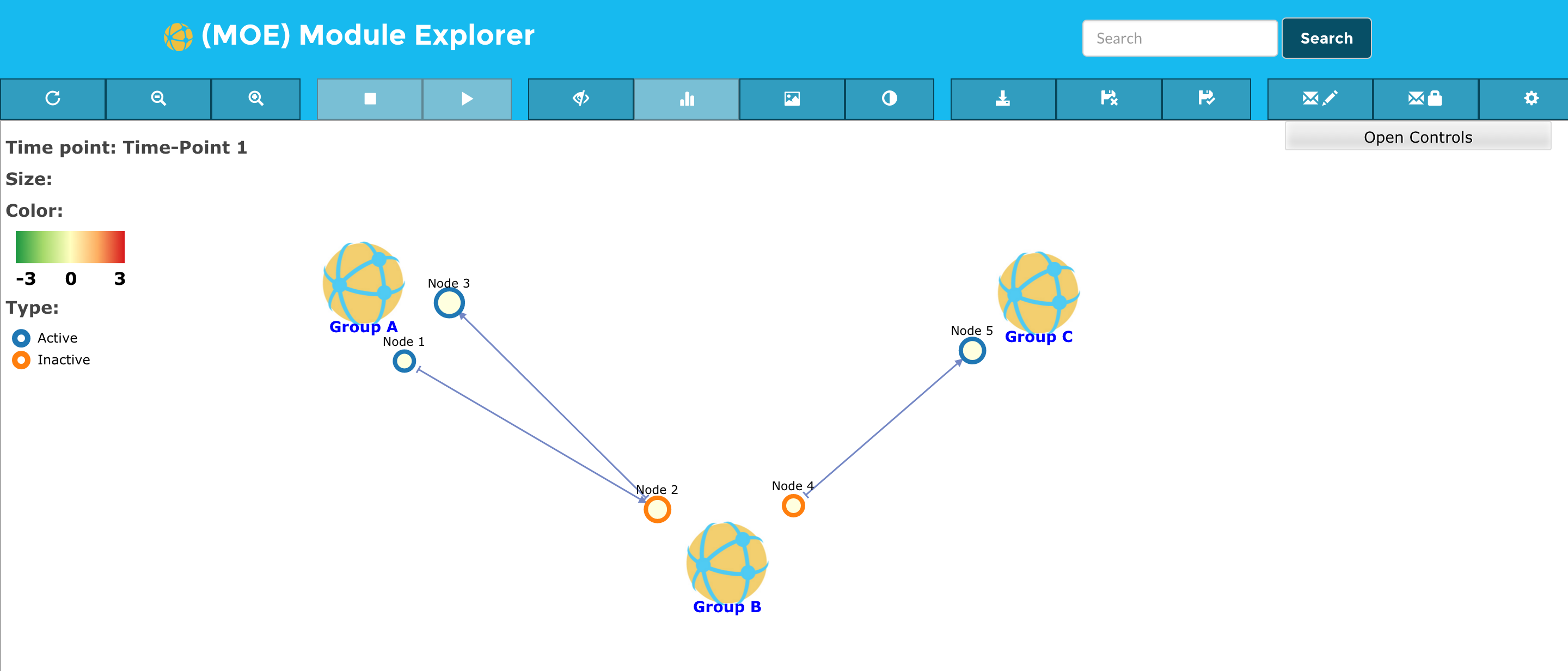


Table 3. PanoromiX Components and Their Functions

| Component | Name | Function / Available Actions |
| --- | --- | --- |
| 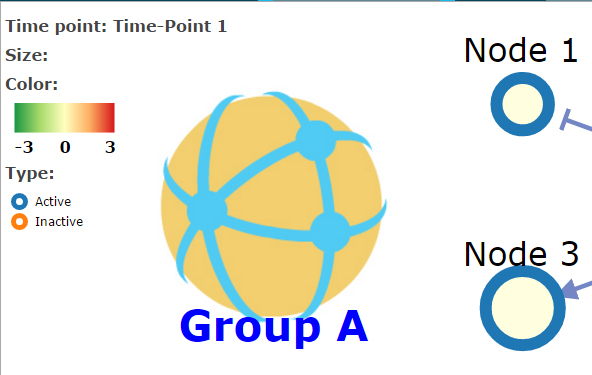  Zoomed In  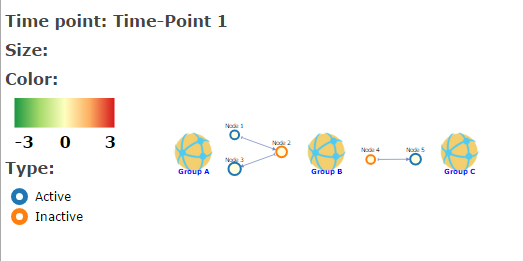  Zoomed Out | Main Visualization Canvas | The visualization canvas displays the entire network with the groups, nodes, and connections that it includes. Using the mouse, you can interact with the canvas in the following ways.  **Action 1: Double-Click / Mouse-Wheel** – When you double-click on an empty area of the canvas, the visualization zooms in by one step. You can also zoom in and out by clicking any of the zoom buttons on the menu bar, using the scroll-wheel on your mouse, or using pinch-gestures for touch-enabled devices or trackpads. This does not affect the size of the legend, as illustrated in the first column of this table.  **Action 2:** **Click & Drag to Pan** – When you click the mouse on an empty area of the canvas and hold down the left mouse button, you can drag the canvas to pan around the network visualization. This is useful for larger networks or to easily navigate while the visualization is zoomed in. Once you release the left mouse button, the visualization remains at the current position and the mouse pointer moves freely without panning. |
| 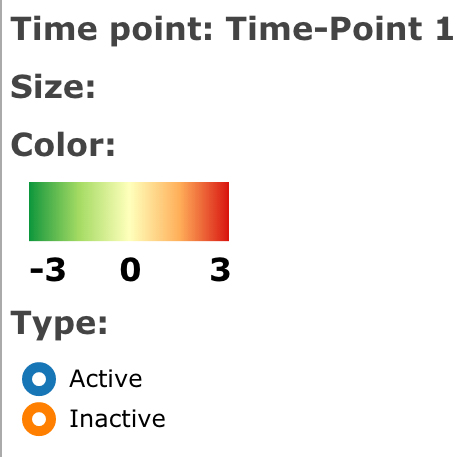 | Legend | The legend shows the current graph labels, color values, and types. Depending on the values you enter for time-point labels, size, and color, you can customize each of these labels *(see more under graph options to follow)*. In addition, you can use options in the PanoromiX dropdown menu to edit the default colors set for type and the color bar gradient, as explained below.  **Action 1:** Mouse-over – When you hover the mouse over any of the listed types, all of the corresponding nodes within the network are highlighted. In this example, the hover is over the “Active” type. |
| 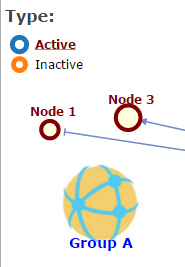  Action 1 |  |  |
| 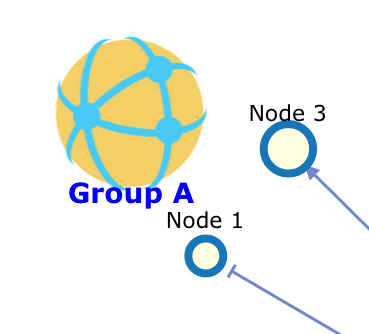 | PanoromiX Node Group & Member Nodes | This represents a group of nodes with the center node bearing the name of the group (Group A) and the sample icon uploaded for the data set. In this case, the center node is surrounded by two nodes in its group, Node 1 and Node 3.  **Action 1:** Mouse-Over – When you hover the mouse over any of the nodes, they increase in size and become highlighted. The node type in the legend also highlights the corresponding node. In this example, we have hovered over “Node 4”.  **Action 2:** Click & Drag – Click and drag any of the group center nodes to another position on the canvas by holding down the left mouse button over the desired node. Once this is done, all nodes that are members of that group follow to the same location. If you drag away member nodes, they return to the area of their group node once you release the left mouse button.  **Action 3:** Double-Click – If you double-click any of the nodes, every other node on the canvas fades out and only the clicked node, plus any nodes that are directly connected to it, remain highlighted. In addition, a small window appears that contains additional information *(if specified in the data file)* such as a description for the double-clicked node. |
| 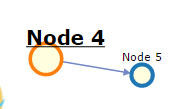  Action 1 |  |  |
| 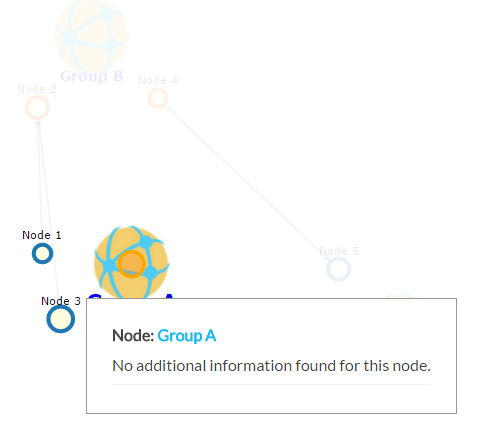  Action 3 |  |  |

### The PanoromiX Toolbar Controls

The following table describes the function of each of the controls that make up the PanoromiX toolbar, which appears at the top of the visualization interface. There are 15 buttons separated into 5 groups: Zoom Controls, Animation Controls, Visualization Options, Save & Download Controls, and Sharing Controls & Graph Label Options.

Table 4. PanoromiX Toolbar Controls

| Button / Control | Name | Function |
| --- | --- | --- |
| 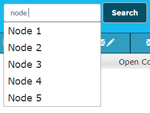 | Search Bar | The search bar allows you to type the name of a particular node in your network and have the visualization locate this node and automatically highlight and center it on the stage. The search has been enhanced with smart-search technology to return an active list of partial matches as you type. Begin typing, then click the name you need *(as seen on left)*. |
| Zoom Controls | | |
| 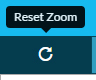 | Reset Zoom | This button resets the zoom level of the network visualization, effectively taking the size of all elements back to the original state that they were first rendered. *(excluding the legend and visualization controls)*. |
| 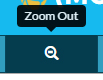 | Zoom Out | This button decreases the zoom level of the network visualization, effectively decreasing the size of all elements *(excluding the legend and visualization controls).* |
| 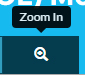 | Zoom In | This button increases the zoom level of the network visualization, effectively increasing the size of all elements *(excluding the legend and visualization controls).* |
| Animation Controls | | |
| 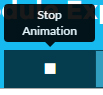 | Stop Animation | While the visualization is currently performing animation through time-points, press this button to stop the animation process at the current state. If you press the start animation button again, it resumes from the current time point. |
| 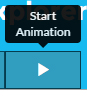 | Start Animation | When time-point data is uploaded to the application, this button displays each time-point, sequentially and in a continuous loop, until you manually stop it using the stop animation button. |
| Visualization Options | | |
| 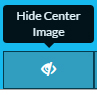 | Hide Center Image | If custom icon images are uploaded for the visualization, this button allows you to hide the icon and display a small circle in its place. Pressing the button again makes the icon visible once more. |
| 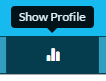 | Show Profile | This button displays a small line chart above each node that represents the uploaded time-series profile data. It shows all of the time series data on this line regardless of the current time-point being displayed. |
| 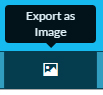 | Export as Image | This button allows you to export the entire visualization at its current state of customization and position. The file generated is a high-resolution PNG image that can be used for many purposes including publication, printing etc. |
| 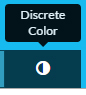 | Discrete Color | This button allows you to switch the type of colors used to represent the nodes. By default, the node type colors are generated using a gradient color system. This button assigns a discrete color for each color value. |
| Save & Download Controls | | |
| 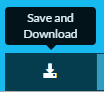 | Save & Download | This button allows you to save the current layout of the project and download a text file *(config.txt)* containing the configuration information that you can later re-upload along with the data files to restore the saved version. |
| 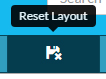 | Reset Layout | This button allows you to reset the graph to its original state when it was first generated by the application. This clears all color changes, customizations, and manual positioning of graph elements. |
| 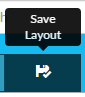 | Save Layout | This button allows you to save the current layout and customization of the visualization. This prevents a reset in case the page is refreshed in the browser or if the workspace link is shared or opened via another computer or browser session. |
| Sharing Controls & Graph Label Options | | |
| 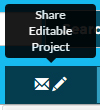 | Share Editable Project | This button opens an additional window that displays a URL that the user can copy and share via email. This option includes the 6-digit key that is generated and allows the recipient to edit, download, and save the project workspace. See Figure 1. |
| 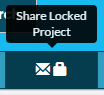 | Share Locked Project | This button opens an additional window that displays a URL that you can copy and share via email. This option does not allow the recipient to edit, download, or save the project workspace as it will not include the authorization key mentioned above. See Figure 2. |
| 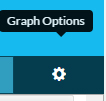 | Graph Options | This button displays a window that allows you to customize each label for your visualization. You can specify type, size, color, and time-point labels. The text you enter is updated live on the visualization interface. See Figure 3. |

For our example data set, the windows described in the table above follow:

Figure 1. Share Editable Project Window


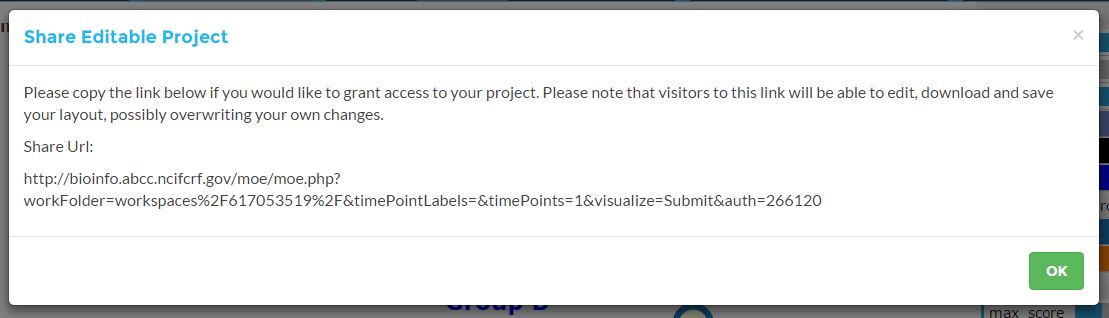


Figure 2. Share Locked Project Window


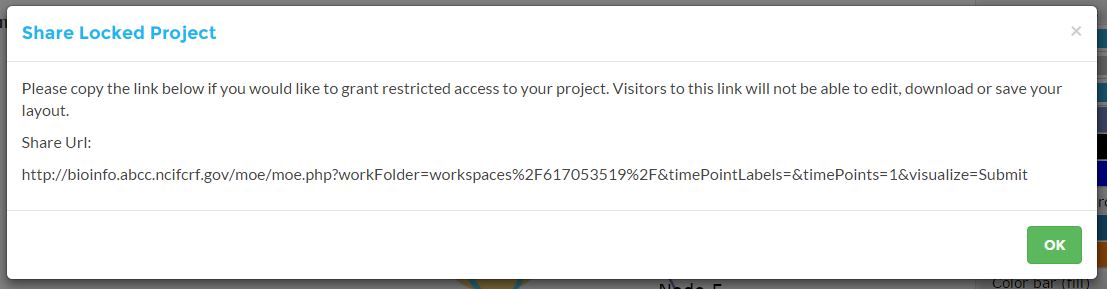


Figure 3. Project Settings Window


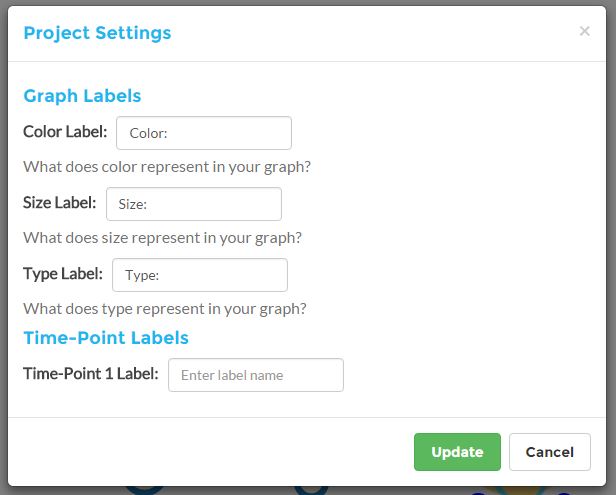


### The PanoromiX Dropdown Controls

This section describes the function of each of the controls that make up the PanoromiX dropdown controls that appear on the right of the visualization interface. For our sample set of data, these controls are separated into three groups: Graph Controls, Type Colors (Stroke), and Color Bar (Fill). Clicking any of the control labels allows you to either collapse individual groups or open/close the entire panel.

| Controls | Name | Function |
| --- | --- | --- |
| Graph Controls | | |
| 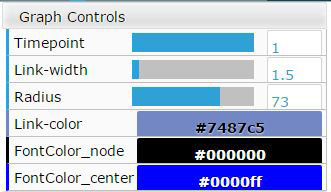 | Timepoint | For data sets with more than one time-point, this allows you to either click-and-drag the mouse to select the point, or enter the numerical value in the textbox to jump there. |
|  | Link-width | This control manipulates the width of the links between nodes. |
|  | Radius | This control manipulates the distance of nodes from their group center icon or node. |
|  | Link-color | This allows you to select or enter the color of links. |
|  | FontColor_node | This controls the color of the node labels. |
|  | FontColor_center | This controls the color of the font used for the center node or group icon labels. |
| Type Colors (Stroke) | | |
| 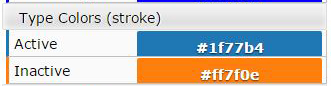 | Active | This control allows you to manipulate a type color. These increase in number according to the different types included in your data. |
|  | Inactive | This is another control for type color. We only have two in our data. |
| Color Bar (Fill) | | |
| 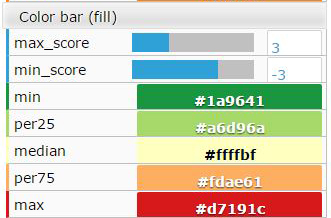 | max_score | This controls the max score number on the color bar gradient. |
|  | min_score | This controls the min score number on the color bar gradient. |
|  | min | This controls the actual color of the minimum value on the color gradient. |
|  | per25 | This controls the actual color of the 25% value on the color gradient. |
|  | median | This controls the actual color of the median value on the color gradient. |
|  | per75 | This controls the actual color of the 7 value on the color gradient. |
|  | max | This controls the actual color of the maximum value on the color gradient. |

## Customizing, Saving, Sharing, and Exporting Your Visualization

Example: Network visualization generated from the sample data

### Using the PanoromiX Controls to Customize Our Network

This section describes a simple customization of the graph, editing the placement of the groups, and then changing colors of, sharing, and exporting our visualization to an image in PNG format. The default rendering of the network follows.


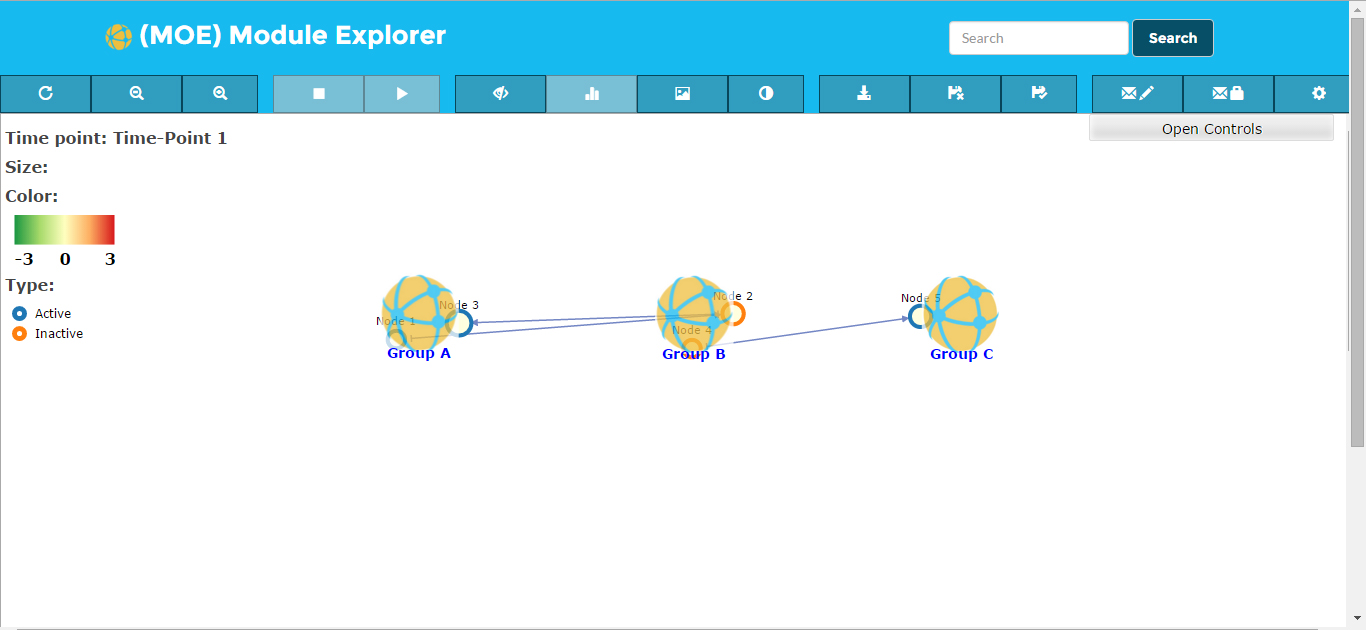


1. Re-arrange the groups by clicking and dragging the center nodes to change their position as shown below:


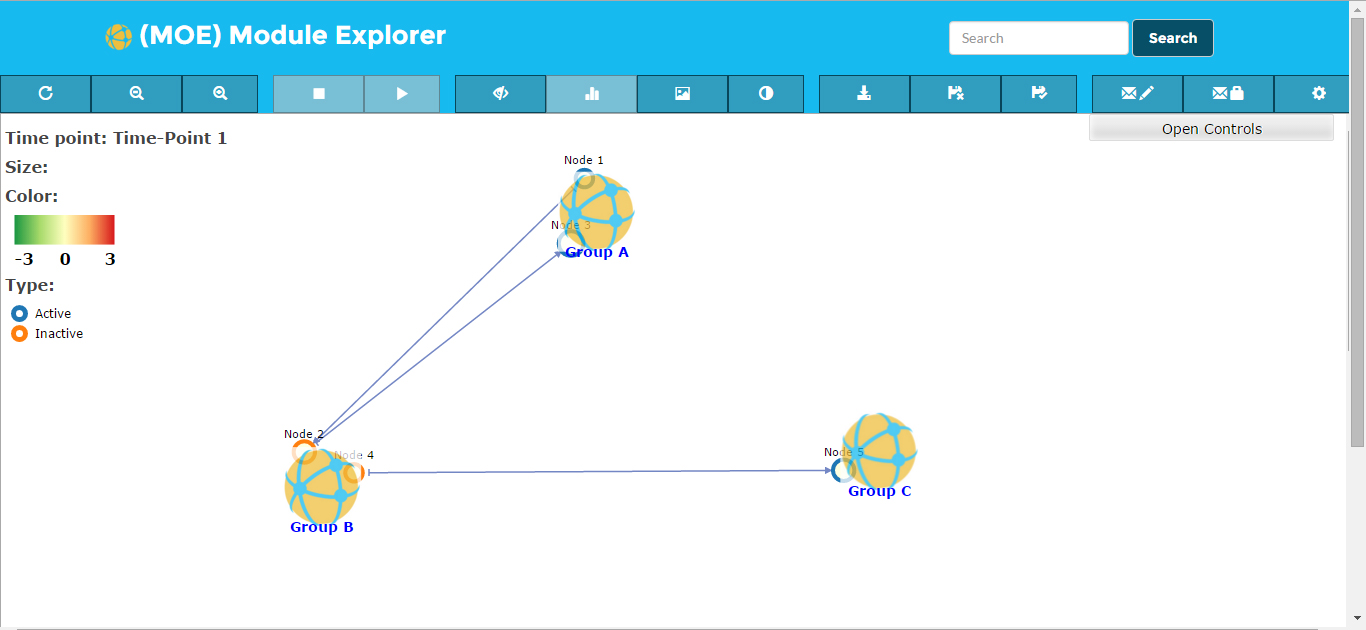


1. Increase the radius, increase the link-width, and change the link and type colors by clicking once on the **Open Controls** drop-down menu on the top right of the canvas, then selecting each option with the mouse and clicking or dragging to the desired value.


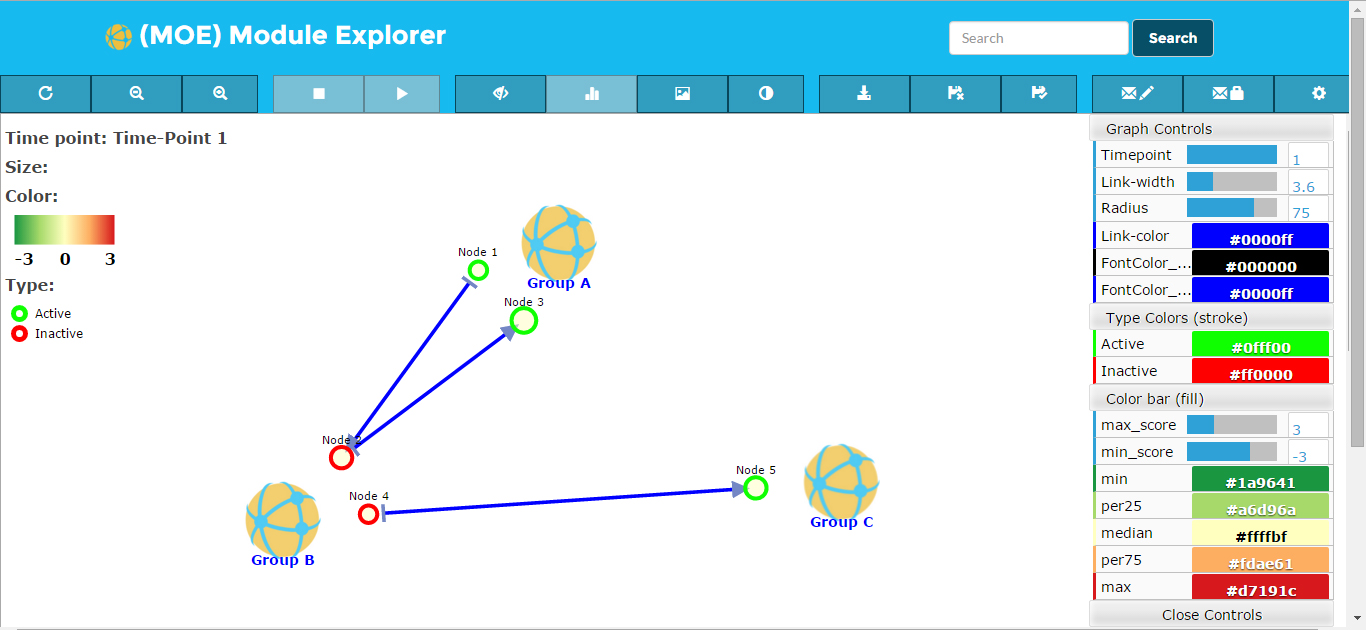


1. Adjust the color bar values for max_score, min_score, min, per25, median, per75, and max by selecting each option with the mouse and clicking or dragging to the desired value. Click the **Hide Center Image** button in the horizontal toolbar menu (
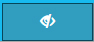
 ) to hide the custom icon you uploaded for the center of each group. Note that it has changed color to indicate that it has been activated and now the center images have been replaced by small orange markers.


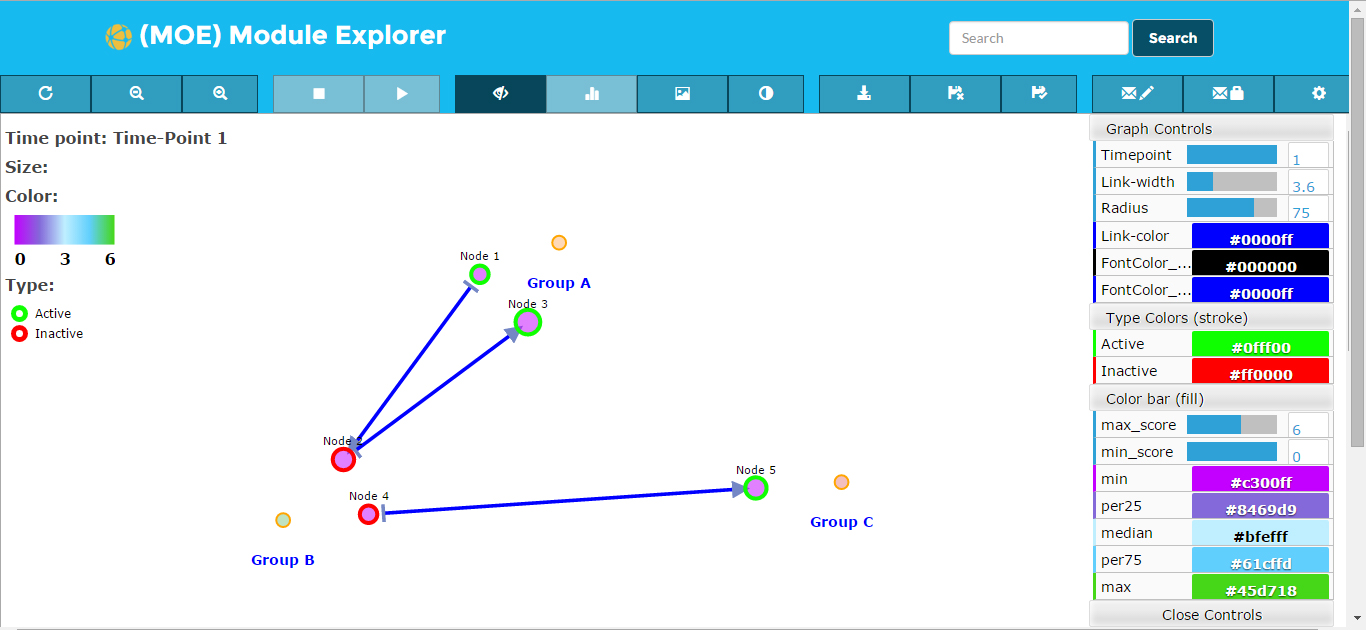


1. Assign values to the graph labels by clicking the **Graph Settings** button

(
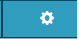
 ) and typing some values in, then clicking the **Update** button. Click the **Hide Center Image** button *(See step 3)* once more to restore the center image icon.


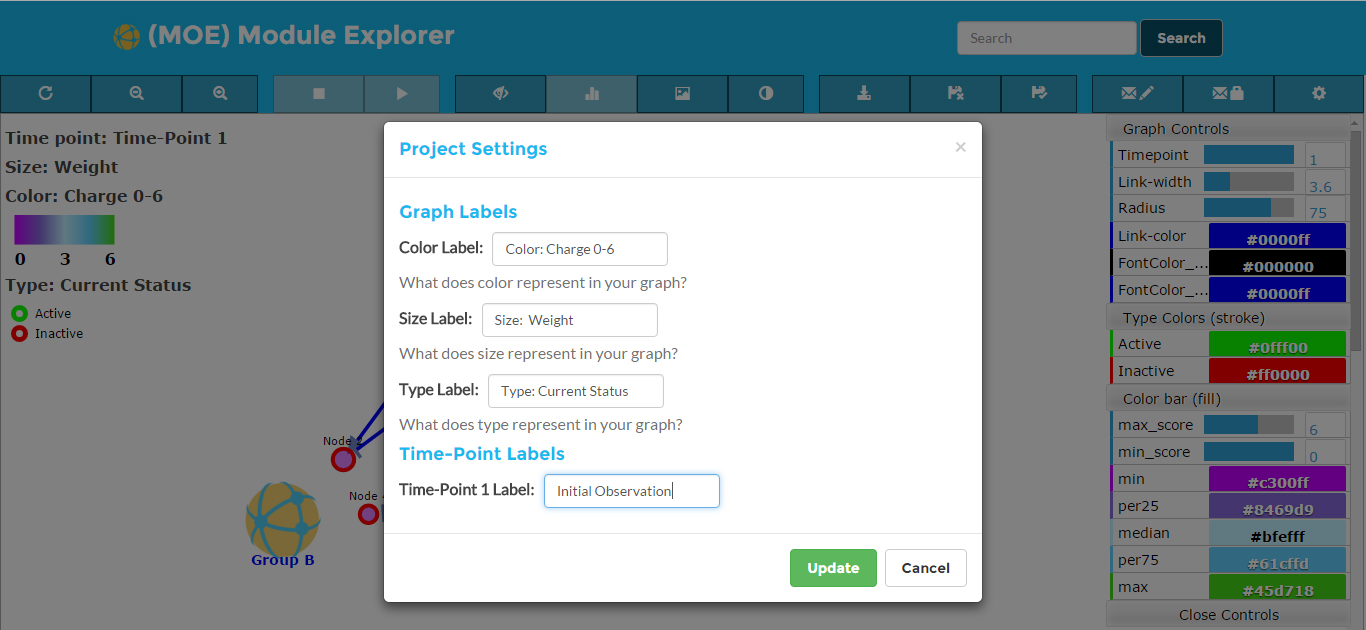


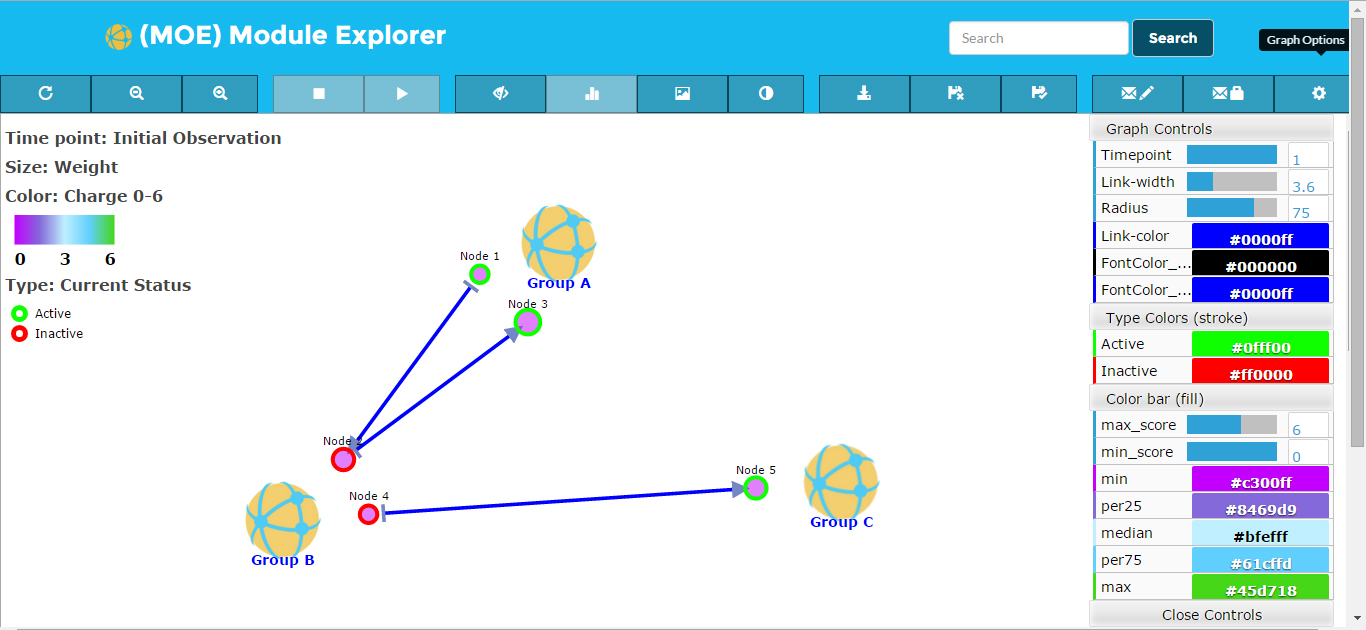


Now that we have customized our graph, save the layout using the **Save Layout** button in our horizontal toolbar.

A small window appears and indicates s that the project layout has been successfully saved. It also provides an authorization key.

“Chapter 3: Advanced Features of PanoromiX,” on page 30, describes in more detail how this key may be used. Record the key in a safe place.


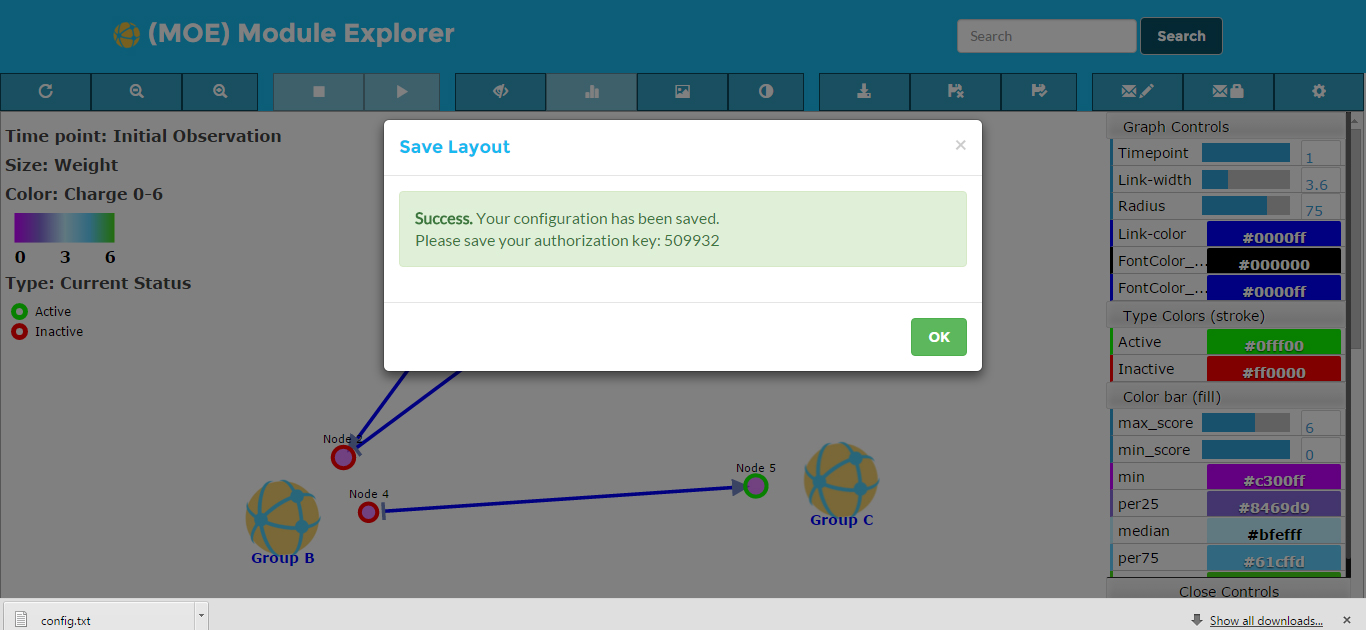


1. The PanoromiX application allows you to share a direct link to the application with others. Decide if you intend for the recipient to modify and save your project once they access it, or if you prefer them to have read-only access. A small window will appear and present you with the *Share URL*. You must copy this link manually and paste it into an email or any other application you want to use to share it. Any recipient of the link would then click it and receive your completely rendered network visualization. See examples for both options below:

NOTE: *Share Locked Project* – the recipient will be able to interact with the network, modify it to their liking and export results to image format only without the ability to save and overwrite your configuration.

*Share Editable Project* - the recipient can modify, download, save and reset the layout of your project in addition to overwriting your saved project configuration.


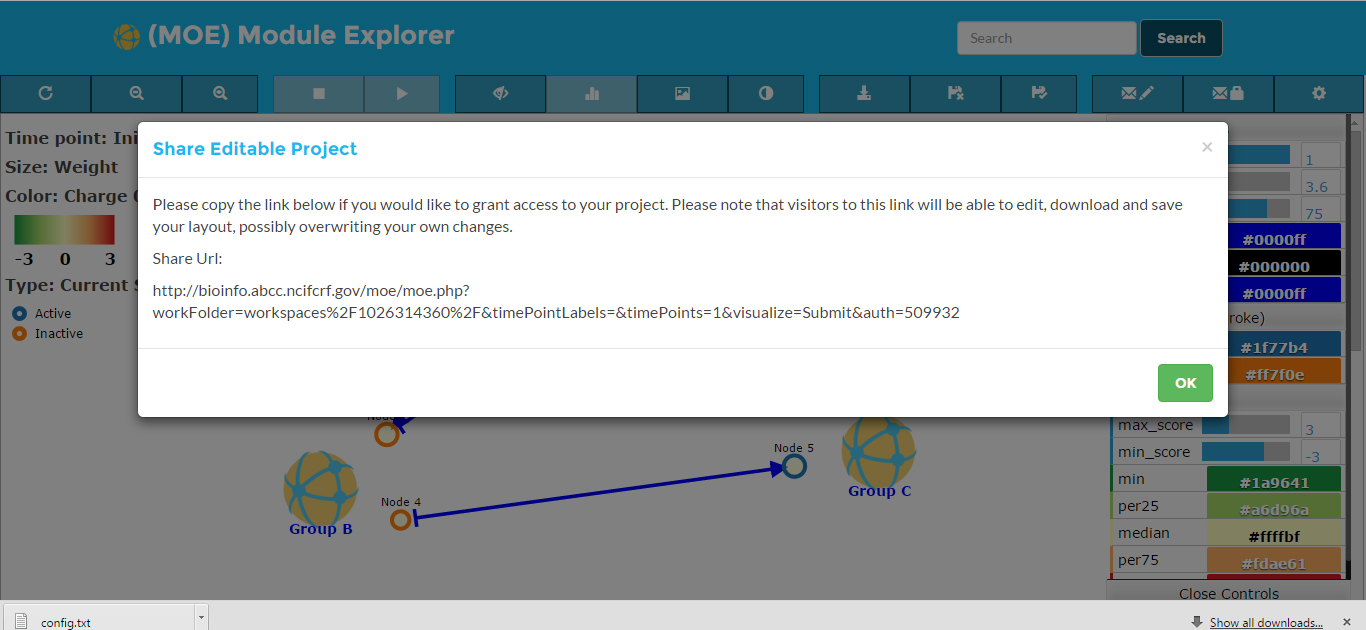


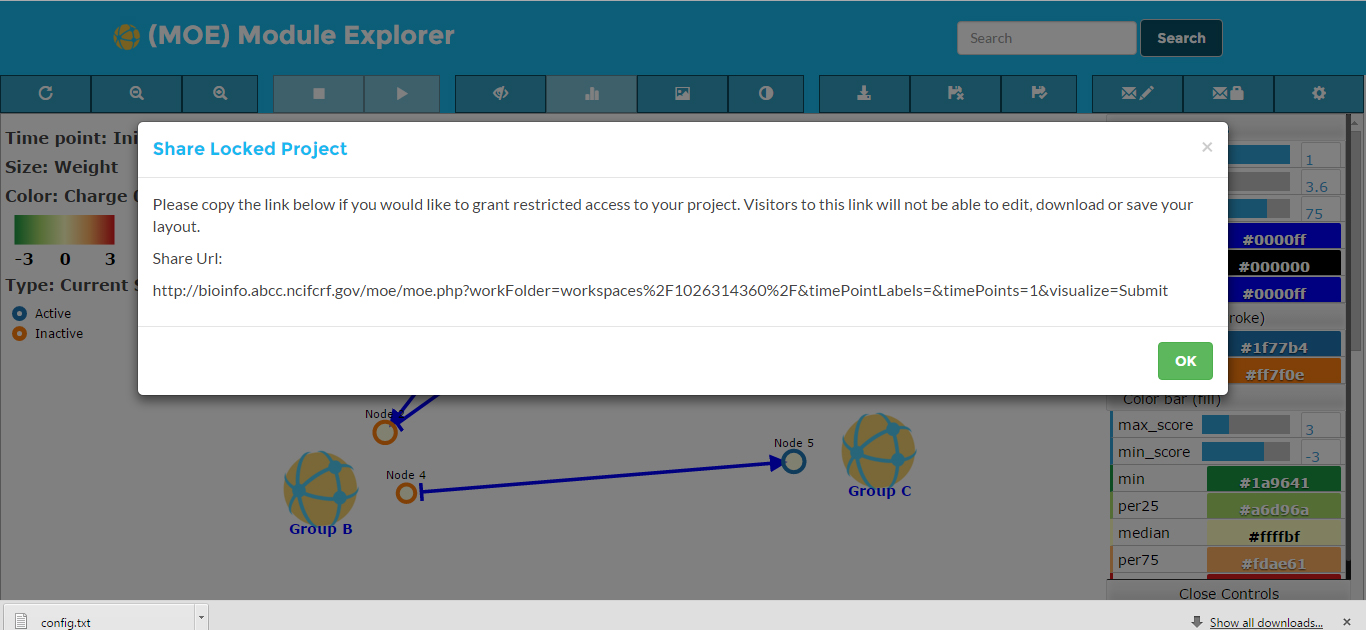


1. In the horizontal toolbar, click the **Export as Image** button to export your network visualization as an image.

   This feature prompts you to save the image in PNG image format with the default filename *PanoromiX-export.png*. You can change the filename but the format will always be the same. You can also see an example exported image saved in JPG format. No cropping is necessary.


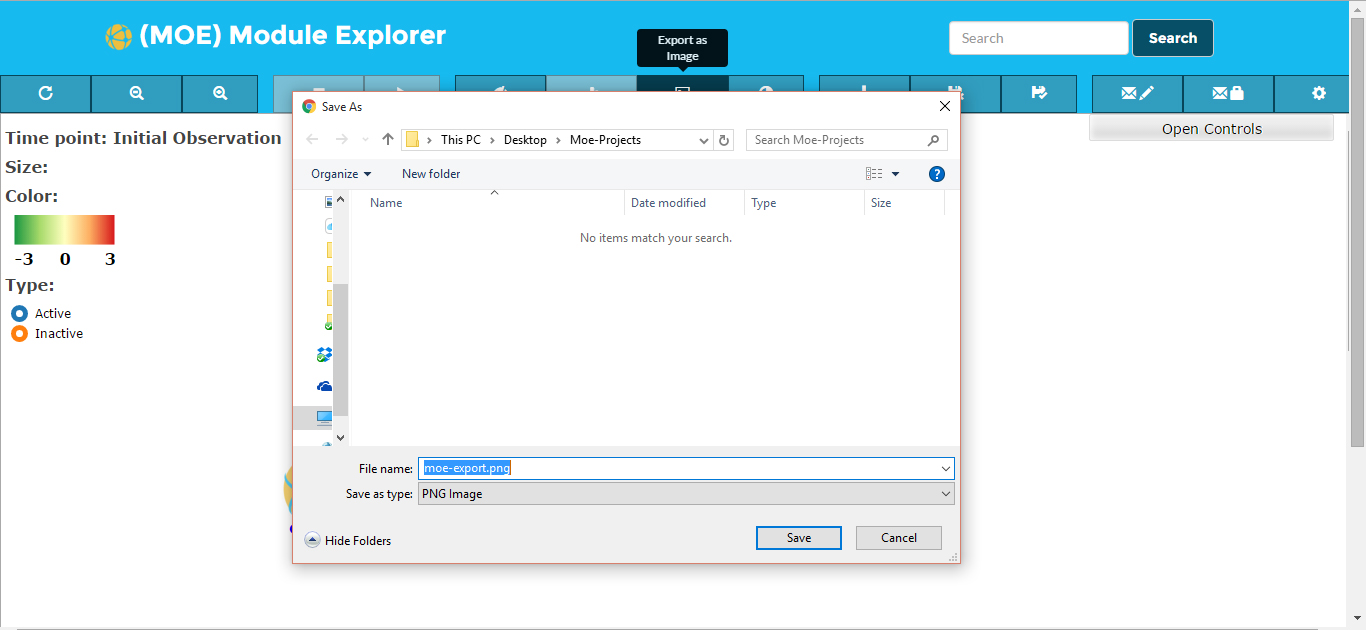


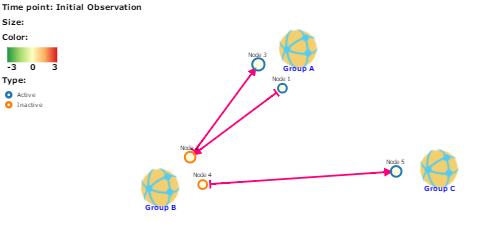


Chapter 3: Advanced Features of PanoromiX

We have covered all of the necessary steps required to create the data files for your project, upload them to the PanoromiX application, customize, and even share and export your project.

Here we will explain the following advanced features of the application:

- Uploading a previously saved PanoromiX project (on this page)
- Including time-point data for animations in your project (on page 31)
- Including additional information: node shapes, descriptions for your project (on page 32)

## Uploading a Previously Saved PanoromiX Project

Example: Using the sample project we created in Chapter 2

In the previous chapter, we outlined the steps necessary to upload data files, create, customize and save a PanoromiX project. Since it is possible to share a project via URL, it is also possible to download a configuration file of a saved PanoromiX project layout and then re-upload this file at a later time, along with the original data, to recreate the saved PanoromiX layout.

The steps for uploading a saved configuration are exactly the same as for creating a new PanoromiX project except for a few steps:

1. Once you have created a PanoromiX project as explained in Chapter 2, click the **Save and download** **project** button (
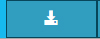
 ). This prompts you to download a text file called *config.txt* that contains all of the layout and customization information. You will use this to re-upload the project along with your data files later, to recreate the PanoromiX network layout.
2. Once you have downloaded your *config.txt* file for your project, proceed to re-upload this file along with your data by making selections in Step 2 of the upload process:

**Step 2. Did you already save or create a configuration for a PanoromiX project? -** This checkbox allows you to upload a configuration file from a previously saved PanoromiX project. Check this box and you will now be presented with an additional upload module. Select
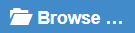
 and navigate to the location of your saved *config.txt* file.

**
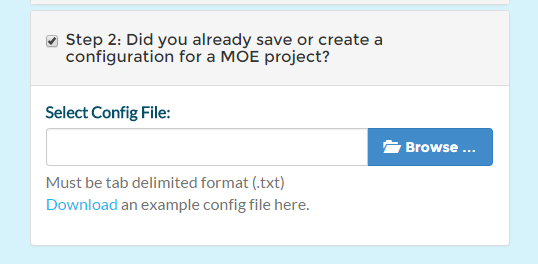
**

1. As long as you have performed this step, proceed to upload the nodes file and links file and optional icon images as previously shown, and the PanoromiX application re-creates the network visualization layout as you had saved it during the download project stage.

## Including Time-Point Data for Animations in Your Project

Example: Modifying the sample data we uploaded in Chapter 2

In addition to visualizing your uploaded data, the PanoromiX application provides the ability to upload information on various time points along with your original data file, and provide you with an animated display of the network as the information associated with nodes, groups and links changes in real time.

Taking the original sample data file as a base, proceed to add the following fields and values so that the new data file appears as follows.


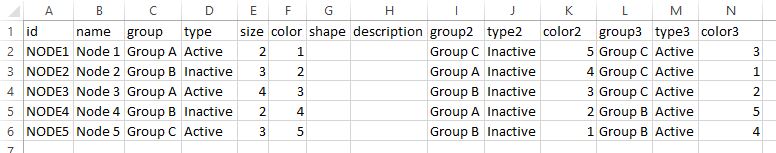


In this way, we have provided a different set of values for a second group and type, for example, which the application will use to adjust the rendering as it displays a looping animation through the various time points we have defined. In this case, since there are three separate groups, types, and also added values for color, this creates three different time point values for the application. During the upload, we can indicate that we have included time-point values in our data and provided labels and other details for the application:


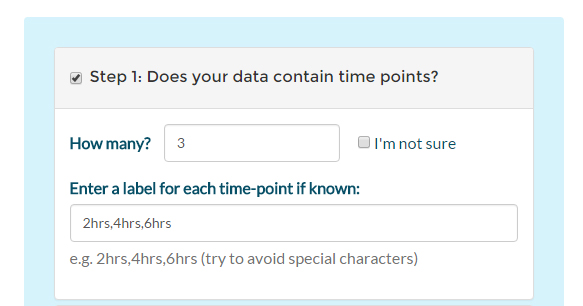


With the added data, once the application renders the visualization, we can now view the color profile value by clicking the **Show Profile** button, which displays the color profile beside each node as shown below.


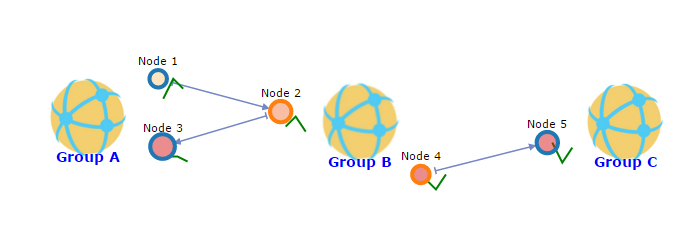


To show the time-point animation, click the **Start Animation** button once, and the application automatically loops through all available time points. To pause or stop the animation at its current position, click the **Stop Animation** button. In the dropdown controls menu, you can also use the mouse to click between specific time points or even type a numeric value in for the specific time point you want to view.

## Including Additional Information: Node Shapes, Descriptions

Example: Modifying the sample data uploaded in Chapter 2

Using the modified data from the step above, add node descriptions and shape information, to further customize your visualization.


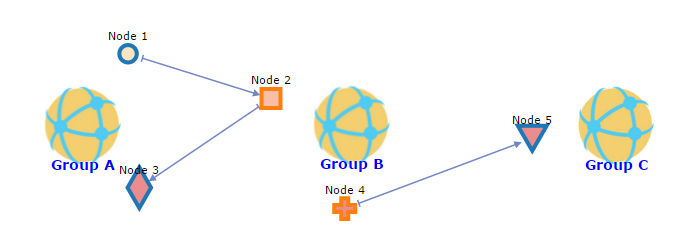


In addition to customized node shapes, we have also added descriptions so that when you double click a node, the descriptive information appears in a small window.


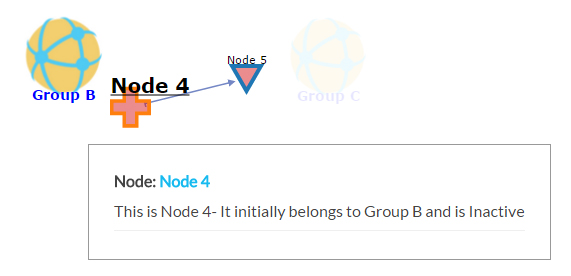


Chapter 4: Frequently Asked Questions and Troubleshooting

## Frequently Asked Questions

### What format does my data need to be in for the PanoromiX application?

All uploaded data must be tab-delimited text with a *.txt* file extension. If you are saving this data from Microsoft Excel, be sure to follow these guidelines:

- From Mac (OSX) versions of MS Excel, save the data file as *Windows Formatted Text* to avoid an error on upload.
- From Windows, save as *Text (Tab-Delimited)*. This applies to both the nodes and the links files.

### What hardware/software do I need to install to run the PanoromiX application?

Since the application is web-based, the only hardware and software requirements are a PC or Mac with an active internet connection and capable of running a recent web browser with JavaScript enabled (most are by default). For best results we recommend Google Chrome or Mozilla Firefox. PanoromiX is also compatible with Internet Explorer, Safari and other web browsers.

### How can I prepare my images for uploading as icons?

For best results, ensure that your images are relatively square in shape so as to avoid any misalignment issues. You are also required to upload only jpeg files with the file extension *.jpg* that are less than 5MB in size. When naming your icons, be certain to match the filename exactly to that of the group you want it to represent. For example, for an icon to represent Group A, it must be named *Group A.jpg*.

### How can I share my PanoromiX project with a collaborator?

You can easily grant access to your PanoromiX project by using one of the two sharing methods. You can copy a link to a locked project, which does not allow the recipient to save or download your project, or you can copy a link to an editable version of your project, which will allow the recipient full control over it, as you would have. Sharing is as easy as copying the provided URL and pasting it in the body of an email or other message you will send. The recipient then clicks the link they receive from you, and they are navigated directly to your visualization. This link is valid for exactly two weeks from the date the project was created.

### How can I export my project as an image for publication or printing?

To export your network for use in documents, publications or printing, click the *Export as Image* button and you will be prompted to download the network as a PNG image file. This file can then be opened and manipulated in any image processing application if necessary before use. We recommend the open source image editor GIMP as a free solution for image editing, cropping and rescaling.
More information here: <https://www.gimp.org/>.

### Can I save my project and return to it at a later time to continue my work?

Absolutely, there are two ways to accomplish this. The easiest method is to click the **Save Layout** button after you customize your project, and bookmark the link. This link will be valid for exactly two weeks and will be stored on our servers for the duration of that time after which the project will be removed. If you want to retain a PanoromiX project for a long term use or backup, click the **Save and Download** button. This will provide you with a saved configuration of your project which you may re-upload along with your data files at a later date, to restore your project for another two-week period. You may repeat this process as many times as you want.

## Troubleshooting

### I am receiving errors while uploading my data, what should I do?

Ensure that you are following the error message generated by the application and that you follow the data template exactly as provided or outlined in the user manual. All fields in your data must be as shown and care must be taken when saving your data that it must be text in tab-delimited format. Save as Text (Tab-Delimited) from MS Excel on Windows, and as Windows Formatted Text when saving from Mac OSX.

### I am having difficulty with some of the application options, or some options are not working for me.

Ensure that you are using a web-browser with JavaScript enabled. This is required for the PanoromiX application to function. Also, some users have reported difficulty with the Export as Image feature while using the Safari browser. For best results, we recommend using either Google Chrome or Mozilla Firefox, both of which are free downloads and compatible with either PC or Mac.

Google Chrome: <http://www.google.com/chrome>

Mozilla Firefox: <https://www.mozilla.org/en-US/firefox/new/>

### One of my recipients is having difficulty opening a shared link to my project.

Ensure that you are copying the entire link from the share project window. Try opening another browser window and visiting the link you copied before you share it just to be sure it is functional. We do not recommend copying and pasting the link directly from your browser address bar as this may grant your recipient unintended editable access to your project.

### My icon images are not being displayed. What can I do?

Pay close attention to the file names used for your icons. They are case sensitive and must be exactly matching the group you wish them to identify. This includes spaces and other characters you may have within your group names. Avoid special characters such as (#%&/.:;!@) etc. Underscore (_) and Dashes (-) are permitted.

### I have uploaded my data but the visualization canvas is blank and does not display my network. What can I do?

Review your data to ensure you have followed the sample data fields format, ensure you have not included special characters in your data values, ensure you have saved your files in the correct format from the application of choice and that you have uploaded the correct files to the correct slot (that is, nodes file to nodes slot and optional links file to links slot). If these steps do not resolve your issues, please contact the PanoromiX support team for additional assistance.

If you encounter issues not addressed by this user guide, please contact the PanoromiX support team for additional support.
